# Modeling schistosomiasis transmission: the importance of snail population structure

**DOI:** 10.1186/s13071-021-04587-8

**Published:** 2021-02-03

**Authors:** Larissa C. Anderson, Eric S. Loker, Helen J. Wearing

**Affiliations:** 1 Department of Biology, University of New Mexico, Albuquerque, NM 87131 USA; 2 Department of Mathematics and Statistics, University of New Mexico, Albuquerque, NM 87131 USA

**Keywords:** Schistosomiasis, Mathematical modeling

## Abstract

**Background:**

Schistosomiasis is a neglected tropical disease endemic in 54 countries. A major* Schistosoma* species, *Schistosoma mansoni*, is sustained via a life cycle that includes both human and snail hosts. Mathematical models of *S. mansoni* transmission, used to elucidate the complexities of the transmission cycle and estimate the impact of intervention efforts, often focus primarily on the human host. However, *S. mansoni* incurs physiological costs in snails that vary with the age of the snail when first infected. Snail demography and the age of snail infection could thus affect the force of infection experienced by humans, which is frequently used to predict the impact of various control strategies.

**Methods:**

To address how these snail host and parasite interactions influence model predictions, we developed deterministic models of schistosomiasis transmission that include varying complexity in the snail population age structure. Specifically, we examined how model outputs, such as schistosome prevalence in human and snail populations, respond to the inclusion of snail age structure.

**Results:**

Our models suggest that snail population age structure modifies the force of infection experienced by humans and the relationship between snail infection prevalence and corresponding human infection prevalence. There are significant differences in estimated snail infection, cercarial density and mean worm burden between models without snail population dynamics and those with snail populations, and between models with a homogeneous snail population and those with age stratification. The variation between finely age-stratified snail populations and those grouped into only juvenile and adult life stages is, however, minimal.

**Conclusions:**

These results indicate that including snails and snail age structure in a schistosomiasis transmission model alters the relationship between snail and human infection prevalence. This highlights the importance of accounting for a heterogeneous intermediate host population in models of schistosomiasis transmission where the impact of proposed control measures is being considered.
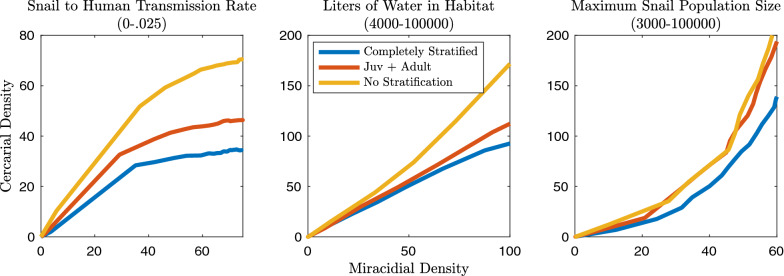

## Background

Schistosomiasis is a neglected parasitic disease caused by various trematode species of the genus *Schistosoma,* for which 218 million people needed treatment in 2015 [1]. Chronic schistosomiasis infections can lead to serious complications such as kidney damage and enlargement of the liver and spleen [2]. One of the most common* Schistosoma* species, *Schistosoma mansoni*, is dependent on both humans and specific species of planorbid snails within the genus *Biomphalaria* for sustained transmission and survival. However, modeling efforts are frequently focused solely on the human host and typically neglect the non-linear dynamics that may be occurring within the snail segment of the transmission cycle. Because this parasitic life cycle has proved robust to elimination, a re-examination of the key assumptions of mathematical models and their consequences for informing control interventions is warranted.

There is a long tradition of the application of mathematical modeling to problems of schistosomiasis control. Numerous mathematical models of schistosomiasis transmission have addressed heterogeneity in infection risk in humans due to spatial factors, socioeconomic status, immune competency and behavior [3–10]. There have been fewer studies that address the role of heterogeneity in the intermediate host population for *S. mansoni* transmission. These studies have incorporated spatial distribution of snails, the prepatent infection period, differential mortality, logistic population growth and parasite-induced castration [3–10]. However, models often classify the snail population as relatively homogeneous, discounting the role that age structure of intermediate hosts could play in disease dynamics.

Age structure within definitive hosts has been shown to impact transmission, and for some diseases, to change the basic reproductive number (*R*_0_) and estimates of the impact of proposed control measures [11–14]. For instance, age population structure has been found to alter *R*_0_ estimates for rubella, influenza and other diseases [15, 16]. Broadly, host age effects are due to age-specific differences in contact patterns, host immune competency, susceptibility and transmission capability, and fitness costs [12, 17–24]. Overall, significantly less work has centered on the consequences of age structure in intermediate host populations. However, population age structure has been shown to be important in models of *Echinococcus* transmission. In that system, age structure is relevant because the parasite maturation is fast relative to the intermediate host lifespan and there is differential infection load and output between intermediate host age classes [25]. There are distinct parallels between that system and the *Biomphalaria* and *S. mansoni* interaction with respect to infection persistence in the intermediate host and the relationship between intermediate host age and parasite output.

*Schistosoma* infection in the intermediate host incurs an array of fitness costs. These costs include: reduced fecundity and the eventual castration of the snail, increased rates of mortality, and in certain life stages a reduction in growth rate [26–28]. The consequences of infection may then play a role in the structuring of the snail population and its growth rate. Conversely, the age of the snail at the time of infection can also impact the relative success of the parasite. The quantity of cercariae produced by the infection of a single snail is dependent on the snail surviving the prepatent period, its subsequent lifespan and the size of snail [29, 30]. In general, the greater the snail size the greater the gonadal tissue volume available for cercarial production within the snail host [31]. As schistosomiasis infection can decrease the lifespan and growth rate of the intermediate host, the timing of infection may impact the lifetime cercarial output of the snail. There are several competing fitness costs associated with *S. mansoni* infection, which may lead to interactions between snail and human host infection levels that are not immediately obvious or intuitive.

In the present work, we assess the effect of age structure within the intermediate host population on the potential force of infection (FOI) to humans. The FOI is an integral component of mathematical models and is used to estimate the impact of treatment interventions on overall schistosomiasis prevalence [32–38]. Estimates of the FOI are usually extrapolated from human or snail prevalence data [27, 37]. In models without the inclusion of snails, the FOI is assumed to be directly (and linearly) related to the human infection prevalence or overall population egg output [33]. Herein we show that FOI estimates may vary widely depending on the inclusion of snails in the model and the age structure profile of the snail host population.

## Methods

### Mathematical model

We developed a deterministic model framework, formally described by a system of ordinary differential equations, which allows us to study the impact of snail population structure on schistosomiasis transmission. Compartments within the mechanistic model include schistosome, cercarial and miracidial life stages and definitive human hosts, and allow us to consider intermediate hosts of several age groups. The human population is kept constant in size as schistosomiasis results in morbidity but not in significant mortality in humans; it is also kept constant in size to isolate the effects of snail dynamics. Humans can have a worm burden of zero, a prepatent infection or a worm burden corresponding to a light, moderate or heavy infection. We also explore an alternative model that utilizes mean worm burden instead of infection intensity, the results of which are presented in the Additional file 1. These infection intensity designations are based on the World Health Organization’s classification system and relate directly to the number of schistosome eggs found per gram of stool in infected individuals [1]. The snail hosts can be susceptible to schistosome infection, exposed and in a pre-patent stage of infection, or infectious. Infection in humans lasts on average 3 years, after which they are again susceptible to reinfection without any additional immune protection [39]. Acquired immunity to schistosomiasis following infection has been extensively debated: reinfection is common, and any acquired immunity may only be partially protective and take a long period of intense exposure to develop; therefore, human immunity is not included within this model [40–42]. Within the snail population, *S. mansoni* infection is assumed to be lifelong.

**Fig. 1a, b:**
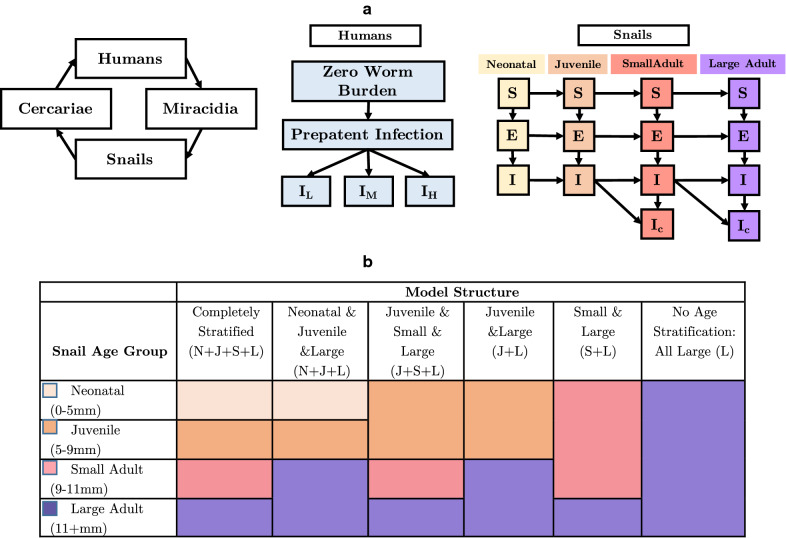
Model structure diagrams. **a** Diagrams of the model framework illustrating the complete transmission cycle, the human infection status with variable *Schistosoma mansoni* worm burden, and the age-stratified snail population.* S* Susceptible class,* E* exposed class,* I* infected class,* I*_*C*_ infected and castrated snails;* I*_*L*_ infected humans with a low *S. mansoni* burden,* I*_*M*_ infected humans with a moderate *S. mansoni* burden,* I*_*H*_ infected humans with a heavy *S. mansoni* burden. **b** The completely stratified model for the snail population includes neonatal (*N*), juvenile (*J*), small (*S*) and large (*L*) adult age classes; this is simplified in various combinations in later model structures. The* colors* represent the way in which the age classes are combined in each model structure. The missing age groups in all models, excluding the completely stratified model, are subsumed into the larger age class. For example, in the neonatal and juvenile and large (*N*+*J*+*L*) model, small adults are subsumed into the large adult age class. In the small and large (*S*+*L*) model, neonatal and juvenile snails are subsumed into the small adult snail class. In every version of the model the snails can still be assigned to any of the infection statuses

We consider seven different model structures, six of which represent varying complexity in snail age structure and one model without the inclusion of any snails in the *S. mansoni* transmission model (Fig. 1). This level of detail is included to determine if non-linear feedback between human and snail infection predicted to occur with fine-scale snail age stratification, due to the age-specific fitness costs, is significantly different enough between population structure options to warrant the added complexity of the inclusion of all age groups. This is of particular interest as parameterizing detailed snail age structure from field studies may be time intensive and logistically difficult.

Within the completely stratified model, snail age groups include neonatal, juvenile, small adult and large adult life stages. These correspond to specific *Biomphalaria glabrata* shell size classes of 0–5 mm, 5–9 mm, 9–11 mm and 11+ mm, respectively. The size classes have been shown in laboratory studies to have varying fitness responses to infection and differential schistosome cercarial shedding after infection [5, 29, 30, 43–45]. All model versions include fitness costs of infection. These fitness costs include: reduced growth rate, reduced or arrested fecundity, and increased mortality with infection. Snails infected in either the neonatal or juvenile class have slowed growth rates and therefore slowed progression into the adult classes [5, 24]. Snails that are infected in either adult class do not display diminished growth. Increased mortality after infection is consistent across all age classes. If infection with *S. mansoni* occurs prior to reproductive maturity then reproduction does not occur; after reproductive age is reached castration of the snail occurs after approximately 6 weeks [46]. These age categories and their corresponding snail shell size represent differing cercarial output post-*S. mansoni* infection [5, 27, 29, 30, 47].

Conceptual diagrams of the completely stratified and alternative model structures are portrayed in Fig. 1. In general, the merged groups are considered to have the averages of the characteristics and parameters associated with the component groups and weighted by the time spent in each age group. For instance, the juvenile and small and large (J+S+L) model has effectively three age classes: juvenile, small adult and large adult, as the neonatal age group is subsumed into the juvenile age group. The merged neonatal and juvenile group will have a parameter that is a weighted average of the neonatal and the juvenile values. The model without snail age groups assumes all snails are large adults; this is a common assumption in other models, as large snails are more likely to be collected in field surveys. A full description of the model parameters is found in Table 1, and a full description of all model subset equations can be found in the supplementary materials.

**Table 1:** Description of model parameters and ranges used in the Latin hypercube parameter sets. All parameter values correspond to *Schistosoma mansoni* and *Biomphalaria glabrata*

Parameter name	Parameter description	Parameter range	References
*m*	Maximum number of snails in the environment	5000–20,000	Estimated
*c*	Liters of water in the environment	1000–100,000	Estimated
*l*_2_	Reduced rate of maturation in infected neonates/juveniles	0.714 of maturation rate in uninfected neonates/juveniles	[35, 38]
*l*_3_	Averaged reduced rate of maturation in infected snails in the combined neonate/juvenile/small adult class	0.75 of maturation rate in uninfected snails	[35, 38]
*w*_1_	Average time to mature from neonate to juvenile	7 weeks	[35, 38, 51]
*w*_2_	Average time to mature from juvenile to small adult	5 weeks	[35, 38, 51]
*w*_3_	Average time to mature from small adult to large adult	2 weeks	[35, 38, 51]
1/*d*_*m*_	Lifespan of miracidia	4–16 h	[10]
1/*d*_*c*_	Lifespan of cercariae	8–20 h	[10]
*u*_1_	Mortality rate of uninfected neonatal snails	0.007–0.152/week	[35, 36]
*u*_2_	Mortality rate of infected neonatal snails	0.062–0.607/week	[35, 36]
*u*_3_	Mortality rate of uninfected juvenile snails	0.007–0.152/week	[35, 36]
*u*_4_	Mortality rate of infected juvenile snails	0.062–0.607/week	[35, 36]
*u*_5_	Mortality rate of uninfected small adult snails	0.007–0.152/week	[35, 36]
*u*_6_	Mortality rate of infected small adult snails	0.062–0.607/week	[35, 36]
*u*_7_	Mortality rate of uninfected large adult snails	0.007–0.152/week	[35, 36]
*u*_8_	Mortality rate of infected large adult snails	0.062–0.607/week	[35, 36]
1/*u*_9_	Lifespan of humans	60 years	[48]
*f*_1_	Fecundity of susceptible small adult snails	40/week	[26, 29, 30, 46, 49]
*f*_2_	Fecundity of exposed small adult snails	4.77/week	[5, 29, 46, 49, 50]
*f*_3_	Fecundity of infected but not castrated snails	4.77/week	[5, 29, 30, 46, 49, 50]
*f*_4_	Fecundity of susceptible large adult snails	40/week	[5, 29, 30, 46, 49, 50]
*f*_5_	Fecundity of exposed large adult snails	4.77/week	[5, 29, 30, 46, 49, 50]
*f*_6_	Fecundity of infected but not castrated adult snails	4.77/week	[5, 29, 30, 46, 49, 50]
*f*_7_	Weighted average fecundity of susceptible snails in the combined neonatal/juvenile/small adult class	13.33/week	[5, 29, 30, 46, 49, 50]
*f*_8_	Weighted average fecundity of exposed or infected but not castrated snails in the combined neonatal/juvenile/small adult class	1.59/week	[5, 29, 30, 46, 49, 50]
*g*	Castration rate of snails	0.1667/week	[46]
1/*y*	Prepatent period in snails	18–45 days	[39]
*e*_1_	Exposure rate of neonatal snails to miracidia	50–150/week	Estimated
*e*_2_	Exposure rate of juvenile snails to miracidia	50–150/week	Estimated
*e*_3_	Exposure rate of small adult snails to miracidia	50–150/week	Estimated
*e*_4_	Exposure rate of large adult snails to miracidia	50–150/week	Estimated
*p*_1_	Per parasite susceptibility for neonatal snails	0–1	[5, 51, 52]
*p*_2_	Per parasite susceptibility for juvenile snails	0–1	[5, 51, 52]
*p*_3_	Per parasite susceptibility for small adult snails	0–1	[5, 51, 52]
*p*_4_	Per parasite susceptibility for large adult snails	0–1	[5, 51, 52]
*s*_1_	Shedding rate of neonatal snails	2800/week	[29, 43]
*s*_2_	Shedding rate of juvenile snails	5500/week	[29, 43]
*s*_3_	Shedding rate of small adult snails	6000/week	[29, 43]
*s*_4_	Shedding rate of large adult snails	6800/week	[29, 43]
*s*_5_	Averaged shedding rate in combined neonatal/juvenile snail class	3925/week	[29, 43]
*s*_6_	Averaged shedding rate in combined small/large adult snail class	6774/week	[29, 43]
*s*_7_	Averaged shedding rate in combined neonatal/juvenile/small adult snail class	4925/week	[29, 43]
*b*	Snail-to-human transmission rate	0.00001–0.001/week	Estimated
*h*_*w*_	Probability of eggs reaching water and hatching	0–1	[27, 39, 51]
*q*_1_	Recovery rate among lightly infected humans	0.2–0.333/year	[39]
*q*_2_	Recovery rate among moderately infected humans	0.2–0.333/year	[39]
*q*_3_	Recovery rate among heavily infected humans	0.2–0.333/year	[39]
1/*l*_*h*_	Latent period in humans	25–30 days	[39]
*v*_1_	Proportion of infected humans who are lightly infected	0–1	Estimated
*v*_2_	Proportion of infected humans who are moderately infected	0–1	Estimated
*v*_3_	Proportion of infected humans who are heavily infected	0–1	Estimated
*g*_1_	Eggs produced by a lightly infected human	7000/week	[39, 53]
*g*_2_	Eggs produced by a moderately infected human	28000/week	[39, 53]
*g*_3_	Eggs produced by a heavily infected human	70000/week	[39, 53]
*g*_4_	Eggs produced per mated female schistosome	350 /day	[54]
*i*	Grams of feces produced per human	200/day	[55]
*N*_*h*_	Total human population size	200	Estimated
*z*	Scaling parameter	1–10	Estimated
*k*	Worm aggregation constant	0.24	[51]

### Model simulations and output

**Fig. 2:**
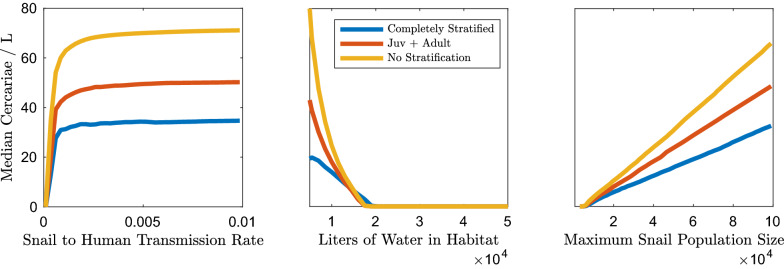
Global sensitivity analyses using Latin hypercube sampling (LHS) parameter sets and isolating the impact of changes in median cercarial density to force of infection (FOI) and snail density. The median cercarial density is primarily sensitive to the density of snails with high levels of infection possible only with high snail density, irrespective of the snail-to-human transmission rate. There are greater levels of cercarial density for the model without snail age stratification than for models with snail age stratification at comparable snail density and snail-to-human transmission rate values.* Juv* Juvenile

The seven model structures (detailed in Fig. 2) were explored to discover the impact of varying levels of complexity in age structure. The metrics calculated to evaluate the effect of age structure include: snail and human infection prevalence, the relationship between these infection levels at equilibrium, cercarial density, mean worm burden, the ratio of cercarial and miracidial density at equilibrium, and the *R*_0_ of the transmission system. The snail-to-human transmission rate was estimated and represents a compound rate of weekly exposure given cercarial density and the probability of infection given exposure. Multiplied by the density of cercariae in the environment, this represents the FOI experienced by humans. In models without snails, the FOI is the product of the transmission rate and the miracidial density. Mean worm burden was calculated by using estimates of eggs produced per mated pair of worms per day, eggs produced per gram of feces in each human infection class, grams of feces produced per human per day and the set number of humans in our system [1, 54, 55]. The classic Anderson and May mean worm burden model was also used for comparison and is presented in Additional file 1: Figures S18 and S19 [39]. The system *R*_0_ values for age class combinations were obtained through next generation matrix methods [18, 56]. The *R*_0_ sensitivity analyses, simulation results, and next generation matrix equations are fully described in the Additional file 1. All model simulations were performed in (MATLAB r2019; The MathWorks, Natick, MA).

### Sensitivity analyses

In order to account for the potential variability in parameter values, two types of sensitivity analyses were performed. First, we performed local sensitivity, varying each parameter value while keeping all others constant. These sensitivity analyses determined the model parameters with the most impact on the key model outcomes of interest: human infection prevalence, mean worm burden, snail infection prevalence, cercarial density, and *R*_0_ of the entire system. The resulting line plots for those parameters found to alter our model outcomes are presented in the supplementary information (Additional file 1: Figures S1–S5). The interaction of parameters that were found to impact the outcomes of interest was further explored by the generation of heat maps. The interaction of the snail-to-human transmission rate and the snail density, both estimated parameters, and their impact on cercarial density, snail and human infection levels and mean worm burden are shown in Additional file 1: Figures S6–S9. The remaining heat maps are presented in the supplementary materials (Additional file 1: Figures S10–S12).

Second, we performed global multivariate sensitivity analyses using Latin hypercube sampling (LHS) for the 44 parameters presented in Table 1. Those parameters that represented probabilities were uniformly sampled within the range 0–1. In the specific case of the proportion of infected humans the total proportion was set to 1. Parameters not restricted to values between 0 and 1 were sampled from uniform distributions within the empirically determined ranges presented in Table 1. Parameters with only a single estimate from the literature or a minuscule range were sampled from a range 0.5–2 times that presented in Table 1. The sensitivity of the transmission system to any estimated parameters is explored over an extensive range. Due to the large number of parameters and the paucity of data, LHS was used to compile 10,000 possible parameter sets to gain confidence in the relationships between these variables and snail and human infection prevalence over a spectrum of parameter values. These 10,000 possible parameter sets were used for each of the seven model options: completely age stratified; neonatal and juvenile and large adult snails; juvenile and small and large adult snails; juvenile and large adult snails; small and large adult snails; no age stratification—all large adults; snail-less model.

## Results

### Sensitivity analyses

**Fig. 3:**
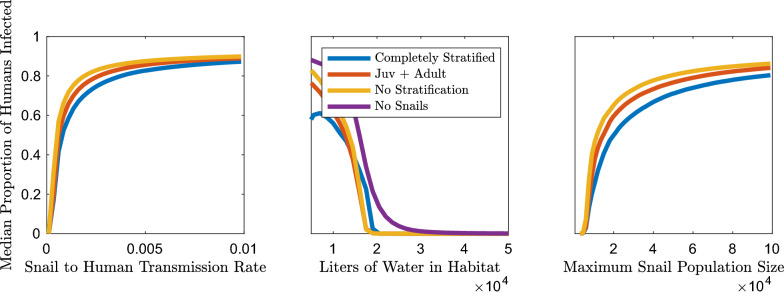
Global sensitivity analyses using LHS parameter sets and isolating the impact of changes in median proportion of humans infected to FOI and snail density. The median proportion of humans infected at equilibrium is sensitive to both the snail-to-human transmission rate, and the density of snails. There are greater proportions of humans infected for the model without snail age stratification than for models with snail age stratification at comparable snail density and snail-to-human transmission rate values. For abbreviations, see Fig. 2

Sensitivity analysis allowed us to identify the parameters that are most influential for the outcomes of interest. The outcomes of interest (*R*_0_, mean worm burden, human and snail prevalence, and cercarial density—which is directly related to the FOI experienced by humans), are impacted by snail density and the snail-to-human transmission rate. The impact of snail density and snail-to-human transmission rate on cercarial density and human infection prevalence are presented in Figs. 2 and 3. The impacts on snail infection prevalence, mean worm burden and *R*_0_ are shown in Additional file 1: Figures S13, S14 and S17. Snail density can be altered in one of two ways, either by modifying the maximum number of snails in the environment or the size of the habitat. These may change through different mechanisms. For instance, maximum snail population size may be impacted by competition, resource availability (which may not be exclusively correlated with habitat size), and snail control measures. Habitat size may fluctuate due to seasonal variation in rainfall or human-mediated habitat alteration.

The relative ranking of unstratified, partially age stratified and completely age stratified for the highest median cercarial densities, holds over a range of snail-to-human transmission rates, habitat sizes and maximum snail populations sizes (Fig. 2). The sensitivity analyses indicate that snail density, as modified by habitat size or maximum snail population size, has an appreciable impact on cercarial density and snail infection prevalence (Fig. 2; Additional file 1: Figure S13). The snail-to-human transmission rate impacts the median cercarial density only in that there is a threshold it needs to exceed for sustained *S. mansoni* transmission, after which there is no appreciable impact on median cercarial density (Fig. 2). Increases in the maximum snail population size lead to linear increases in cercarial density because, with a constant habitat size, the cercarial population is directly related to the absolute quantity of snails in the environment (Fig. 2). The unstratified model displays higher median cercarial density because the cercarial production for an infected snail is related to snail age and size and the unstratified model is comprised of only large adult snails. These large adult snails produce more per capita cercariae when infected than any other snail age class.

The median proportion of snails infected saturates over a range of snail-to-human transmission rate values (Additional file 1: Figure S13). The impact of the snail-to-human transmission rate is directly applied to human infection; however, the effect on snail infection is diluted by the processes occurring in the human to snail portion of the transmission cycle. Similarly, maximum snail population size did not have an appreciable effect on the median proportion of snails infected after a certain snail population size was reached (Additional file 1: Figure S13). Conversely, the habitat size had a substantial impact on snail infection; as the habitat size increased the snail infection levels decreased (Additional file 1: Figure S13). This reduction is due to the decrease in the miracidial and cercarial density with larger water body volume. Common to all of these simulations is the negligible differences in median snail infection levels for unstratified snail age models versus age-stratified models.

In contrast to cercarial density and snail infection prevalence, human infection prevalence and mean worm burden are not only heavily impacted by snail density but show a more nuanced relationship with the snail-to-human transmission rate (Fig. 3; Additional file 1: Figure S14). This disparity is due to the composition of the FOI experienced by humans. The FOI is the product of the snail-to-human transmission rate and cercarial density, which is correlated with snail density, whereas snail infection prevalence is indirectly related to the snail-to-human FOI through the link between human infection prevalence and snail infection prevalence. Similar to snail infection and cercarial density, all model types display decreasing human infection as the habitat size increases (Fig. 3). The increase in habitat size decreases miracidial density, and therefore the human-to-snail FOI, snail density, cercarial density and subsequently the snail-to-human FOI. The maximum snail population size only marginally increases the median proportion of infected humans after a snail population size sufficient for sustaining transmission has been reached. The impact of the snail-to-human transmission rate does saturate as human infection prevalence nears 100%; however, at more moderate levels of human infection prevalence (40–80%) there are disparities between model types. In particular, as with cercarial density, unstratified models predicted higher values than age-stratified model types (Fig. 3).

### Snail infection

**Fig. 4:**
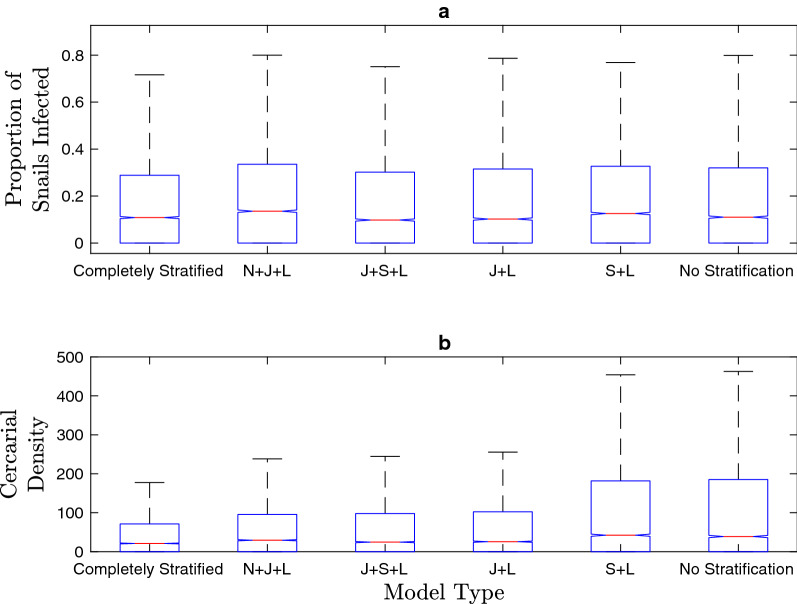
Estimates of **a** snail infection prevalence, and **b** cercarial density for each model subset with constructed possible parameter sets from LHS. **a** All models display comparable median snail infection prevalence. **b** The models with juvenile and adult snail age stratification display a lower median and a narrower range of probable cercarial densities than the models with only adult snails. For abbreviations, see Figs. 1 and 2

Estimates of the proportion of snails infected do not vary considerably between models with snail age stratification (Fig. 4a; Additional file 1: Figure S7). Kruskal-Wallis tests indicated that there were not significant differences in the median snail infection prevalence between the model without stratification and all age-stratified models.

The overall cercarial population size, and cercarial density, which is an integral component of the FOI experienced by the human population, varied between the age-stratified and unstratified models (Fig. 4b). The Kruskal-Wallis tests indicate that the median cercarial densities for the unstratified model and the small and large (S+L) model are significantly different from those of the more age-stratified models. The greater cercarial density for the unstratified model, comprised entirely of large adult snails, and for the S+L model is partially due to the greater cercarial output of small and large adult snails and the compounding effect that this has on the levels of human infection and snail infection levels. However, as in the case of snail infection prevalence, the difference between the completely stratified model and the other models with juvenile and adult age classes is negligible. Specific* p*-values for the Kruskal-Wallis tests for cercarial density, snail infection prevalence, human infection prevalence, mean worm burden and* R*_0_ are presented in Additional file 1: Tables S3–S6.

### Human infection

**Fig. 5a, b:**
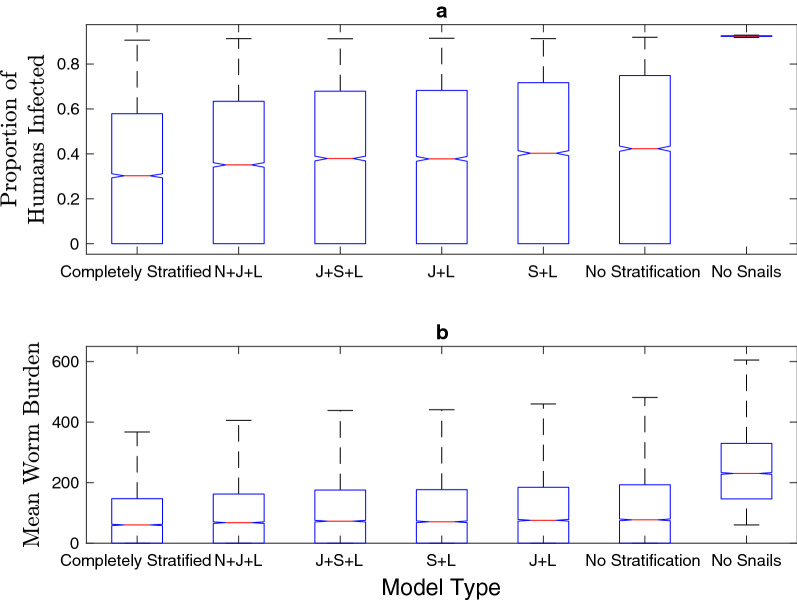
Estimates of median human infection prevalence and mean worm burden for each model subset with constructed possible parameter sets from LHS. The models without any snails or with only adult snails display an expanded range and higher estimates of probable human infection prevalence and mean worm burden than models with any snail age stratification over these ranges. For abbreviations, see Figs. 1 and 2

Similar to predicted cercarial densities, the models without snail age stratification resulted in altered human infection prevalence estimates over a wide range of possible parameter sets, habitat sizes and snail-to-human transmission rate values (Figs. 3, 5a). A model that does not explicitly include the snail population, which assumes a linear feedback between parasite egg output and snail-to-human FOI, also results in human infection levels that differ substantially from models with snail age stratification (Fig. 5a). In the models with a snail population, the snail-to-human FOI is the product of the snail-to-human transmission rate, the rate of infection given exposure, and the density of cercariae in the water, which is assumed to be directly related to exposure risk. Kruskal-Wallis tests indicate that the distribution of the human infection prevalence differed between: models with age stratification including sub-adult classes and the models without sub-adult snail classes (unstratified and S+L), the model without snails and all models including snails. The completely stratified model was different from only the unstratified and model without snails. Within the age-stratified models which include neonatal or juvenile stages none are significantly different from each other.

Mean worm burden mirrors the human infection prevalence trends and Kruskal-Wallis test results because the differences in mean worm burden between the various age-stratified models were minimal (Fig. 5b). We found that mean worm burden as calculated in the method of the Anderson and May model [39] followed the same trends but showed greater differences between the age-stratified models (Additional file 1: Figures S18, S19). It is important to note that the differences in cercarial density, human infection and mean worm burden are quite small between models with snails, and that the overall trends show that any stratification of the snail population that includes juvenile and adult stages appears to be sufficient to capture the variation between unstratified and stratified models.

### Relationship between human and snail infection prevalence

**Fig. 6:**
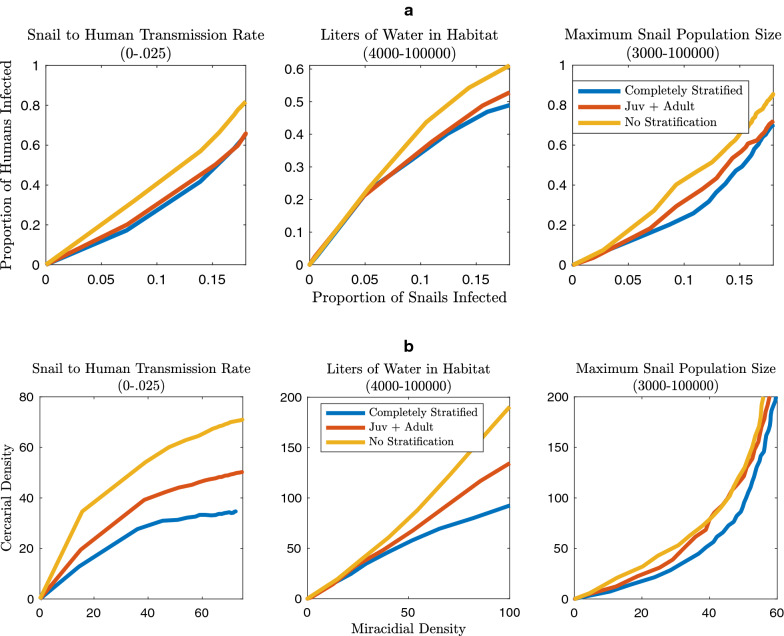
**a** The relationship between snail infection prevalence and human infection prevalence over a large range of snail-to-human transmission rate values, snail populations and habitat sizes. All other parameters are obtained from LHS. The model without snail age stratification estimates higher snail infection prevalence necessary to reach low (0–10%), medium (10–50%) and high (50+%) levels of human infection over a range of FOI and snail densities. **b** The relationship between the total miracidia and cercariae populations over a large range of snail-to-human transmission rate values and snail densities demonstrates a consistent positive relationship between miracidial and cercarial density. For abbreviations, see Fig. 2

The relationship between human infection prevalence and snail infection prevalence over a range of snail-to-human transmission rate values, and maximum snail population sizes is shown in Fig. 6a. There exists a consistent positive correlation between increasing human infection prevalence and snail infection prevalence. This relationship is consistent with the low levels of snail infection and corresponding high levels of human infection found in field studies in endemic areas [57–65]. Over a large range of habitat sizes, increasing snail infection is associated with increasing human prevalence (Fig. 6a). However, low snail infection can be associated with the majority of the range of possible human infection prevalence. There is also minimal disparity between age-stratified and unstratified models over a range of maximum snail population sizes.

### Relationship between cercariae and miracidia

The relationship between miracidial and cercarial density is not consistent over snail-to-human transmission rate values, maximum snail population sizes and habitat volumes. Over a large range of snail-to-human transmission rates, cercarial density saturates with increasing miracidial density, and unstratified models saturate at a higher cercarial density. Equivalent miracidial densities result in higher cercarial density in unstratified models than in age-stratified models. Cercarial density also increases with miracidial density over a range of habitat sizes and unstratified models have higher cercarial densities than stratified models with similar miracidial density values. Over a large range of snail population sizes miracidial density and cercarial density are positively correlated. While there is a difference between the unstratified models and those incorporating snail age, there is a smaller disparity between models including various levels of snail age structure. Overall, the consistency of human infection prevalence, snail infection prevalence, mean worm burden, and of R_0_, between all models with snail age stratification indicates that any stratification that includes both adult and sub-adult groups is sufficient to capture the non-linear dynamics that occur with the inclusion of snails and snail age stratification.

## Discussion

While schistosomiasis-transmission models without explicit snail populations are common in the literature, they may not accurately reflect the range of human infection prevalence or the relationship between snail and human infection prevalence seen in endemic areas [57–61, 63–66]. Accurate estimates of probable changes in snail and human prevalence after reductions in one or both populations are essential to predicting the impact of schistosomiasis control. The changes in predicted FOI and infection prevalence with the inclusion of a snail population indicate that outcomes of major control interest will differ in models which include more detailed snail dynamics. Due to the differences in the feedback between human egg output, FOI, and estimated* R*_0_ values, the inclusion of snail age structure in models could alter the projected impact of proposed control measures.

### Sensitivity analyses

The sensitivity analyses indicate that snail density is a major driver of the outcomes of interest, though the snail-to-human transmission rate is influential in determining the level of human infection. The importance of the snail-to-human transmission rate in this portion of the transmission cycle is due to the fact that the FOI experienced by the human population in this model is a product of cercarial density, which is highly correlated with snail density, and the snail-to-human transmission rate. The effect of the snail-to-human transmission rate may be diluted by the processes occurring between human exposure and snail exposure; the level of human infection and the probability of eggs reaching water sources and successfully hatching.

### Impact of snail age structure

The exclusion of the snail population from the model structure assumes that there is a linear relationship between human egg excretion and the FOI to humans. The differential shedding rates among snails of different ages, and the physiological repercussions of *S. mansoni* infection are predicted to affect the feedback between snail and human infection levels. The results in Figs. 3, 4, 5 support the prediction that the inclusion of snail dynamics in the model influences these outcomes of interest. All of these patterns are consistent for the suite of model simulations run with potential parameter sets produced with LHS.

In our model framework there are two ways to change snail density: alteration of habitat size or maximum snail population size. Changing the habitat size has a direct effect on not only cercarial density but miracidial density as well. Alteration of the maximum snail population size has direct impacts on cercarial density (Fig. 2) but indirect effects on miracidial density. The relationship between cercarial output and miracidial output in this system appears to be linear, with different snail age structure assumptions altering the slope of the linear dependence. However, this linear relationship only holds for lower snail-to-human transmission rates. Over a range of snail-to-human transmission rates, cercariae and miracidia display a saturating relationship as the increased mortality and reduced fecundity associated with *S. mansoni* limits the proportion of snails infected in the population. The greater proportion of large snails in unstratified and S+L model types in comparison to a fully stratified model and their capacity for greater cercarial production is evident in the spread of the median cercarial densities in Fig. 6b. As this cercarial density is directly related to the FOI to humans in these models, human egg output may be robust to fluctuations in the FOI but not changes in habitat size, which may fluctuate seasonally. This is due to the disparity in infection levels between snails and humans; only a small percentage of snails need to be infected and shedding for high levels of human infection to occur. More precise model outcomes could be obtained with additional information on parameter ranges. For instance, the probability of* Schistosoma* eggs reaching water and hatching could be refined with improved estimates on egg viability and hatching rates in the field.

### Implications for control programs

The model output was most sensitive to snail density; however, only relative density of intermediate host species is routinely collected [5]. In order to gain a more precise understanding of this system, it would be prudent to collect information on the absolute density of the intermediate host species. In* Echinococcus* transmission, another intermediate host and parasite system, it is hypothesized that the impacts on definitive host infection level of the intermediate host population age profile could obscure the effects of seasonality on the system as this may be the primary mechanism through which seasonal fluctuations manifest [25]. As snail population size and habitat size may change seasonally it is crucial to understand how the relationship between snail and human prevalence and resulting cercarial and miracidial populations may be altered as these fluctuate. There is evidence that *Biomphalaria* population size varies seasonally, and it is possible that the population age structure shifts seasonally as well [67–69]. Seasonal fluctuations could alter the impact of control measures. Snail density is a crucial measure for the estimation of the FOI for *S. mansoni* transmission models that include snails. However, the underlying forces driving changes in snail density, habitat size and snail population size may result in differing impacts on the transmission cycle.

Further work is needed to quantify the effects of reduction in prevalence in either snail or human infection, as would occur after chemoprophylaxis or molluscicide events, on infection in the other host. It is clear from Fig. 6 that inclusion of snail age structure results in a different relationship between snail and human infection levels than in models without age. However, only minimal age structure needs to be included to capture these differences. With varying snail-to-human transmission rate values, which are often estimated from data, there are disparate estimates of the proportion of snails needed to sustain levels of human infection and vice versa. This may be problematic if those values are used to estimate the impact that a decrease in prevalence for either species would have on the other. For instance, a reduction in snail prevalence from 10 to 5% in a model without snail age stratification would assume a decrease in equilibrium human infection from 75 to 25%, whereas in a model with a fully age-stratified snail population the change would be from 45 to 15%; the same levels of snail infection are assumed to be able to support completely different human infection regimes. Overall, in order to accurately estimate the FOI to humans and the impact of various control strategies, snail demographics should be considered in future modeling efforts.

## Supplementary Information

**Additional file 1.**

## Data Availability

All relevant parameters and model equations are available in the manuscript or in the supplementary material.
