# Supplementary material for: Modeling schistosomiasis transmission: the importance of snail population structure
Source: Parasit Vectors. 2021 Feb 3;14:94. doi: 10.1186/s13071-021-04587-8 (PMC7860629; doi:10.1186/s13071-021-04587-8)
Supplement: Supplementary file 1 — Additional file 1. [file 13071_2021_4587_MOESM1_ESM.pdf]

Supplemental Information

1 Table of Contents

1. Table 1: Description of Model Variables
2. Model Equations: Humans
3. Model Equations: Snails
4. Next Generation Matrix
5. Sensitivity Figures 1 - 14
6. R_0 Figures 15-17
7. Kruskal -Wallis Results Tables 3-6
8. Figure 18: MWB model of Anderson and May Sensitivity
9. Figure 19: MWB model of Anderson and May Boxplots

2 Model Description

Table 1: Description of Model Variables

Variable	Description
S_n	Susceptible neonatal snails
E_n	Exposed neonatal snails
I_n	Infected neonatal snails
S_j	Susceptible juvenile snails
E_j	Exposed juvenile snails
I_j	Infected juvenile snails
S_s	Susceptible small adult snails
E_s	Exposed small adult snails
I_s	Infected small adult snails
I_{sc}	Infected and castrated small adult snails
S_l	Susceptible large adult snails
E_l	Exposed large adult snails
I_l	Infected large adult snails
I_{lc}	Infected and castrated large adult snails
N_{sn}	Total snail population (all ages and infection statuses)
Mir	Miracidia
Cer	Cercariae
S_H	Susceptible humans
E_H	Exposed humans
I_{LH}	Lightly infected humans
I_{MH}	Moderately infected humans
I_{HH}	Heavily infected humans
N_H	Total human population (all infection statuses)

2.1 Model Equations

For all model structures including snails, the differential equations describing the *Human Population* are given by

$$\frac{dS_H}{dt} = u_9 N_H + q_1 I_{LH} + q_2 I_{MH} + q_3 I_{HH} - b\frac{Cer}{c} S_H - u_9 S_H$$
$$\frac{dE_H}{dt} = b\frac{Cer}{c} S_H - l_h E_H - u_9 E_H$$
$$\frac{dI_{LH}}{dt} = v_1 l_h E_H - u_9 I_{LH} - q_1 I_{LH}$$
$$\frac{dI_{MH}}{dt} = v_2 l_h E_H - u_9 I_{MH} - q_2 I_{MH}$$
$$\frac{dI_{HH}}{dt} = v_3 l_h E_H - u_9 I_{HH} - q_3 I_{HH}$$

The differential equations describing the free-living parasite and snail populations differ for each model structure and are detailed below.

2.2 Completely Stratified Model

Neonatal Snail Population

$$\frac{dS_n}{dt} = (f_1 S_s + f_2 E_s + f_3 I_s + f_4 S_l + f_5 E_l + f_6 I_l)\left(1 - \frac{N_{sn}}{m}\right)$$
$$- u_1 S_n - \frac{1}{w_1} S_n - e_1 p_1 \frac{Mir}{c} S_n$$
$$\frac{dE_n}{dt} = e_1 p_1 \frac{Mir}{c} S_n - u_2 E_n - \frac{1}{w_1} E_n - y E_n$$
$$\frac{dI_n}{dt} = y E_n - u_2 I_n - \frac{l_2}{w_1} I_n$$

Juvenile Snail Population

$$\frac{dS_j}{dt} = \frac{1}{w_1} S_n - u_3 S_j - e_2 p_2 \frac{Mir}{c} S_j - \frac{1}{w_2} S_j$$
$$\frac{dE_j}{dt} = \frac{1}{w_1} E_n + e_2 p_2 \frac{Mir}{c} S_j - u_4 E_j - \frac{1}{w_2} E_j - y E_j$$
$$\frac{dI_j}{dt} = \frac{l_2}{w_1} I_n + y E_j - u_4 I_j - \frac{l_2}{w_2} I_j$$

Small Adult Snail Population

$$\frac{dS_s}{dt} = \frac{1}{w_2} S_j - \frac{1}{w_3} S_s - u_5 S_s - e_3 p_3 \frac{Mir}{c} S_s$$
$$\frac{dE_s}{dt} = \frac{1}{w_2} E_j + e_3 p_3 \frac{Mir}{c} S_s - u_6 E_s - \frac{1}{w_3} E_s - y E_s$$
$$\frac{dI_s}{dt} = y E_s - u_6 I_s - \frac{1}{w_3} I_s - g I_s$$
$$\frac{dI_{sc}}{dt} = \frac{l_2}{w_2} I_j + g I_s - u_6 I_{sc} - \frac{1}{w_3} I_{sc}$$

Large Adult Snail Population

$$\frac{dS_l}{dt} = \frac{1}{w_3}S_s - u_7 S_l - e_4 p_4 \frac{Mir}{c} S_l$$
$$\frac{dE_l}{dt} = \frac{1}{w_3}E_s + e_4 p_4 \frac{Mir}{c} S_l - u_8 E_l - yE_l$$
$$\frac{dI_l}{dt} = \frac{1}{w_3}I_s + yE_l - u_8 I_l - gI_l$$
$$\frac{dI_{lc}}{dt} = \frac{1}{w_3}I_{sc} + gI_l - u_8 I_{lc}$$

Miracidia Population

$$\begin{aligned}\frac{dMir}{dt} = {} & h_w(g_1 I_{LH} + g_2 I_{MH} + g_3 I_{HH}) - d_m Mir \\ & - [e_1(S_n + E_n + I_n) + e_2(S_j + E_j + I_j) + e_3(S_s + E_s + I_s + I_{sc}) \\ & + e_4(S_l + E_l + I_l + I_{lc})]\frac{Mir}{c}\end{aligned}$$

Cercariae Population

$$\frac{dCer}{dt} = s_1 I_n + s_2 I_j + s_3(I_s + I_{sc}) + s_4(I_l + I_{lc}) - d_c Cer$$

2.3 Neonatal Juvenile and Large Model

Small and Large Adult Snail Groups Combined

Neonatal Snail Population

$$\frac{dS_n}{dt} = (f_4 S_l + f_5 E_l + f_6 I_l)\left(1 - \frac{N_{sn}}{m}\right) - u_1 S_n - \frac{1}{w_1} S_n - e_1 p_1 \frac{Mir}{c} S_n$$
$$\frac{dE_n}{dt} = e_1 p_1 \frac{Mir}{c} S_n - u_2 E_n - \frac{1}{w_1} E_n - y E_n$$
$$\frac{dI_n}{dt} = y E_n - u_2 I_n - \frac{l_2}{w_1} I_n$$

Juvenile Snail Population

$$\frac{dS_j}{dt} = \frac{1}{w_1} S_n - u_3 S_j - e_2 p_2 \frac{Mir}{c} S_j - \frac{1}{w_2} S_j$$
$$\frac{dE_j}{dt} = \frac{1}{w_1} E_n + e_2 p_2 \frac{Mir}{c} S_j - u_4 E_j - \frac{1}{w_2} E_j - y E_j$$
$$\frac{dI_j}{dt} = \frac{l_2}{w_1} I_n + y E_j - u_4 I_j - \frac{l_2}{w_2} I_j$$

Large Adult Snail Population

$$\frac{dS_l}{dt} = \frac{1}{w_2} S_j - u_7 S_l - e_4 p_4 \frac{Mir}{c} S_l$$
$$\frac{dE_l}{dt} = \frac{1}{w_2} E_j + e_4 p_4 \frac{Mir}{c} S_l - u_8 E_l - y E_l$$
$$\frac{dI_l}{dt} = y E_l - u_8 I_l - g I_l$$
$$\frac{dI_{lc}}{dt} = \frac{l_2}{w_2} I_j + g I_l - u_8 I_{lc}$$

Miracidia Population

$$\frac{dMir}{dt} = h_w (g_1 I_{LH} + g_2 I_{MH} + g_3 I_{HH}) - d_m Mir - [e_1(S_n + E_n + I_n) + e_2(S_j + E_j + I_j) + e_4(S_l + E_l + I_l + I_{lc})] \frac{Mir}{c}$$

Cercariae Population

$$\frac{dCer}{dt} = s_1 I_n + s_2 I_j + s_6 (I_l + I_{lc}) - d_c Cer$$

2.4 Juvenile Small and Large Model

Neonatal and Juvenile Snail Groups Combined

Juvenile Snail Population

$$
\begin{aligned}
\frac{dS_j}{dt} &= (f_1 S_s + f_2 E_s + f_3 I_s + f_4 S_l + f_5 E_l + f_6 I_l)\left(1 - \frac{N_{sn}}{m}\right) \\
&\quad - u_3 S_j - \frac{1}{w_1 + w_2} S_j - e_2 p_2 \frac{Mir}{c} S_j \\
\frac{dE_j}{dt} &= e_2 p_2 \frac{Mir}{c} S_j - u_4 E_j - \frac{1}{w_1 + w_2} E_j - y E_j \\
\frac{dI_j}{dt} &= y E_j - u_4 I_j - \frac{l_2}{w_1 + w_2} I_j
\end{aligned}
$$

Small Adult Snail Population

$$
\begin{aligned}
\frac{dS_s}{dt} &= \frac{1}{w_1 + w_2} S_j - \frac{1}{w_3} S_s - u_5 S_s - e_3 p_3 \frac{Mir}{c} S_s \\
\frac{dE_s}{dt} &= \frac{1}{w_1 + w_2} E_j + e_3 p_3 \frac{Mir}{c} S_s - u_6 E_s - \frac{1}{w_3} E_s - y E_s \\
\frac{dI_s}{dt} &= y E_s - u_6 I_s - \frac{1}{w_3} I_s - g I_s \\
\frac{dI_{sc}}{dt} &= \frac{l_2}{w_1 + w_2} I_j + g I_s - u_6 I_{sc} - \frac{1}{w_3} I_{sc}
\end{aligned}
$$

Large Adult Snail Population

$$
\begin{aligned}
\frac{dS_l}{dt} &= \frac{1}{w_3} S_s - u_7 S_l - e_4 p_4 \frac{Mir}{c} S_l \\
\frac{dE_l}{dt} &= \frac{1}{w_3} E_s + e_4 p_4 \frac{Mir}{c} S_l - u_8 E_l - y E_l \\
\frac{dI_l}{dt} &= \frac{1}{w_3} I_s + y E_l - u_8 I_l - g I_l \\
\frac{dI_{lc}}{dt} &= \frac{1}{w_3} I_{sc} + g I_l - u_8 I_{lc}
\end{aligned}
$$

Miracidia Population

$$
\begin{aligned}
\frac{dMir}{dt} &= h_w (g_1 I_{LH} + g_2 I_{MH} + g_3 I_{HH}) - d_m Mir \\
&\quad - [e_2 (S_j + E_j + I_j) + e_3 (S_s + E_s + I_s + I_{sc}) + e_4 (S_l + E_l + I_l + I_{lc})] \frac{Mir}{c}
\end{aligned}
$$

Cercariae Population

$$
\frac{dCer}{dt} = s_5 I_j + s_3 (I_s + I_{sc}) + s_4 (I_l + I_{lc}) - d_c Cer
$$

2.5 Juvenile and Large Model

Neonatal and Juvenile Snail Groups Combined
Small and Large Adult Snail Groups Combined

Juvenile Snail Population

$$\frac{dS_j}{dt} = (f_4 S_l + f_5 E_l + f_6 I_l)\left(1 - \frac{N_{sn}}{m}\right) - u_3 S_j - \frac{1}{w_1 + w_2} S_j - e_2 p_2 \frac{Mir}{c} S_j$$
$$\frac{dE_j}{dt} = e_2 p_2 \frac{Mir}{c} S_j - u_4 E_j - \frac{1}{w_1 + w_2} E_j - y E_j$$
$$\frac{dI_j}{dt} = y E_j - u_4 I_j - \frac{l_2}{w_1 + w_2} I_j$$

Large Adult Snail Population

$$\frac{dS_l}{dt} = \frac{1}{w_1 + w_2} S_j - u_7 S_l - e_4 p_4 \frac{Mir}{c} S_l$$
$$\frac{dE_l}{dt} = \frac{1}{w_1 + w_2} E_j + e_4 p_4 \frac{Mir}{c} S_l - u_8 E_l - y E_l$$
$$\frac{dI_l}{dt} = y E_l - u_8 I_l - g I_l$$
$$\frac{dI_{lc}}{dt} = \frac{l_2}{w_1 + w_2} I_j + g I_l - u_8 I_{lc}$$

Miracidia Population

$$\frac{dMir}{dt} = h_w(g_1 I_{LH} + g_2 I_{MH} + g_3 I_{HH}) - d_m Mir - [e_2(S_j + E_j + I_j) + e_4(S_l + E_l + I_l + I_{lc})]\frac{Mir}{c}$$

Cercariae Population

$$\frac{dCer}{dt} = s_5 I_j + s_6(I_l + I_{lc}) - d_c Cer$$

2.6 Small and Large Model

Neonatal, Juvenile and Small Adult Snail Groups Combined

Small Adult Snail Population

$$\frac{dS_s}{dt} = (f_7 S_s + f_8 E_s + f_8 I_s + f_4 S_l + f_5 E_l + f_6 I_l)\left(1 - \frac{N_{sn}}{m}\right)$$
$$- u_5 S_s - \frac{1}{w_1 + w_2 + w_3} S_s - e_3 p_3 \frac{Mir}{c} S_s$$
$$\frac{dE_s}{dt} = e_3 p_3 \frac{Mir}{c} S_s - u_6 E_s - \frac{l_3}{w_1 + w_2 + w_3} E_s - y E_s$$
$$\frac{dI_s}{dt} = y E_s - u_6 I_s - \frac{l_3}{w_1 + w_2 + w_3} I_s - g I_s$$
$$\frac{dI_{sc}}{dt} = g I_s - u_6 I_{sc} - \frac{l_3}{w_1 + w_2 + w_3} I_{sc}$$

Large Adult Snail Population

$$\frac{dS_l}{dt} = \frac{1}{w_1 + w_2 + w_3} S_s - u_7 S_l - e_4 p_4 \frac{Mir}{c} S_l$$
$$\frac{dE_l}{dt} = \frac{l_3}{w_1 + w_2 + w_3} E_s + e_4 p_4 \frac{Mir}{c} S_l - u_8 E_l - y E_l$$
$$\frac{dI_l}{dt} = \frac{l_3}{w_1 + w_2 + w_3} I_s + y E_l - u_8 I_l - g I_l$$
$$\frac{dI_{lc}}{dt} = \frac{l_3}{w_1 + w_2 + w_3} I_{sc} + g I_l - u_8 I_{lc}$$

Miracidia Population

$$\frac{dMir}{dt} = h_w (g_1 I_{LH} + g_2 I_{MH} + g_3 I_{HH}) - d_m Mir$$
$$- [e_3(S_s + E_s + I_s + I_{sc}) + e_4(S_l + E_l + I_l + I_{lc})] \frac{Mir}{c}$$

Cercariae Population

$$\frac{dCer}{dt} = s_7 (I_s + I_{sc}) + s_4 (I_l + I_{lc}) - d_c Cer$$

2.7 Unstratified Model

Large Adult Snail Population

$$\frac{dS_l}{dt} = (f_4 S_l + f_5 E_l + f_6 I_l)\left(1 - \frac{N_{sn}}{m}\right) - u_7 S_l - e_4 p_4 \frac{Mir}{c} S_l$$
$$\frac{dE_l}{dt} = e_4 p_4 \frac{Mir}{c} S_l - u_8 E_l - y E_l$$
$$\frac{dI_l}{dt} = y E_l - g I_l - u_8 I_l$$
$$\frac{dI_{lc}}{dt} = g I_l - u_8 I_{lc}$$

Miracidia Population

$$\frac{dMir}{dt} = h_w(g_1 I_{LH} + g_2 I_{MH} + g_3 I_{HH}) - d_m Mir - e_4(S_l + E_l + I_l + I_{lc}) \frac{Mir}{c}$$

Cercariae Population

$$\frac{dCer}{dt} = s_4(I_l + I_{lc}) - d_c Cer$$

2.8 No Snail Model

Miracidia Population

$$\frac{dMir}{dt} = h_w(g_1 I_{LH} + g_2 I_{MH} + g_3 I_{HH}) - d_m Mir$$

Human Population

$$\frac{dS_H}{dt} = u_9 N_H + q_1 I_{LH} + q_2 I_{MH} + q_3 I_{HH} - b\frac{Mir}{c} S_H - u_9 S_H$$
$$\frac{dE_H}{dt} = b\frac{Mir}{c} S_H - l_h E_H - u_9 E_H$$
$$\frac{dI_{LH}}{dt} = v_1 l_h E_H - u_9 I_{LH} - q_1 I_{LH}$$
$$\frac{dI_{MH}}{dt} = v_2 l_h E_H - u_9 I_{MH} - q_2 I_{MH}$$
$$\frac{dI_{HH}}{dt} = v_3 l_h E_H - u_9 I_{HH} - q_3 I_{HH}$$

2.9 Completely Stratified Model with Anderson-May Mean Worm Burden

Neonatal Snail Population

$$\frac{dS_n}{dt} = (f_1 S_s + f_2 E_s + f_3 I_s + f_4 S_l + f_5 E_l + f_6 I_l)\left(1 - \frac{N_{sn}}{m}\right)$$
$$- u_1 S_n - \frac{1}{w_1} S_n - e_1 p_1 \frac{Mir}{c} S_n$$
$$\frac{dE_n}{dt} = e_1 p_1 \frac{Mir}{c} S_n - u_2 E_n - \frac{1}{w_1} E_n - y E_n$$
$$\frac{dI_n}{dt} = y E_n - u_2 I_n - \frac{l_2}{w_1} I_n$$

Juvenile Snail Population

$$\frac{dS_j}{dt} = \frac{1}{w_1}S_n - u_3S_j - e_2p_2\frac{Mir}{c}S_j - \frac{1}{w_2}S_j$$
$$\frac{dE_j}{dt} = \frac{1}{w_1}E_n + e_2p_2\frac{Mir}{c}S_j - u_4E_j - \frac{1}{w_2}E_j - yE_j$$
$$\frac{dI_j}{dt} = \frac{l_2}{w_1}I_n + yE_j - u_4I_j - \frac{l_2}{w_2}I_j$$

Small Adult Snail Population

$$\frac{dS_s}{dt} = \frac{1}{w_2}S_j - \frac{1}{w_3}S_s - u_5S_s - e_3p_3\frac{Mir}{c}S_s$$
$$\frac{dE_s}{dt} = \frac{1}{w_2}E_j + e_3p_3\frac{Mir}{c}S_s - u_6E_s - \frac{1}{w_3}E_s - yE_s$$
$$\frac{dI_s}{dt} = yE_s - u_6I_s - \frac{1}{w_3}I_s - gI_s$$
$$\frac{dI_{sc}}{dt} = \frac{l_2}{w_2}I_j + gI_s - u_6I_{sc} - \frac{1}{w_3}I_{sc}$$

Large Adult Snail Population

$$\frac{dS_l}{dt} = \frac{1}{w_3}S_s - u_7S_l - e_4p_4\frac{Mir}{c}S_l$$
$$\frac{dE_l}{dt} = \frac{1}{w_3}E_s + e_4p_4\frac{Mir}{c}S_l - u_8E_l - yE_l$$
$$\frac{dI_l}{dt} = \frac{1}{w_3}I_s + yE_l - u_8I_l - gI_l$$
$$\frac{dI_{lc}}{dt} = \frac{1}{w_3}I_{sc} + gI_l - u_8I_{lc}$$

Miracidia Population

$$\begin{aligned}\frac{dMir}{dt} = {} & (\phi g_4 hu) - d_m Mir \\ & - [e_1(S_n + E_n + I_n) + e_2(S_j + E_j + I_j) + e_3(S_s + E_s + I_s + I_{sc}) \\ & + e_4(S_l + E_l + I_l + I_{lc})]\frac{Mir}{c}\end{aligned}$$

Cercariae Population

$$\frac{dCer}{dt} = s_1I_n + s_2I_j + s_3(I_s + I_{sc}) + s_4(I_l + I_{lc}) - d_cCer$$

Mean Worm Burden

$$\frac{dMWB}{dt} = bz(Cer) - (u_9MWB)$$

Mating function:

$$\phi = 1 - (\frac{1}{2\pi})\int_0^{2\pi} \frac{(1 - (\cos(\theta))d\theta}{[1 + (1 + \cos\theta)\frac{MWB}{k}]^{k+1}}$$

2.10 Next Generation Matrix

Completely Stratified Model

	$\boldsymbol{E_n}$	$\boldsymbol{E_j}$	$\boldsymbol{E_s}$	$\boldsymbol{E_l}$	$\boldsymbol{Cer}$	$\boldsymbol{E_H}$	$\boldsymbol{Mir}$
$\boldsymbol{E_n}$	0	0	0	0	0	0 0	$Mir \to E_n$
$\boldsymbol{E_j}$	0	0	0	0	0	0	$Mir \to E_j$
$\boldsymbol{E_s}$	0	0	0	0	0	0	$Mir \to E_s$
$\boldsymbol{E_l}$	0	0	0	0	0	0	$Mir \to E_l$
$\boldsymbol{Cer}$	$E_n \to Cer$	$E_j \to Cer$	$E_s \to Cer$	$E_l \to Cer$	0	0	0
$\boldsymbol{E_H}$	0	0	0	0	$Cer \to E_H$	0	0
$\boldsymbol{Mir}$	0	0	0	0	0	$E_H \to Mir$	0

Table 2: The ij^{th} cell of the Next Generation Matrix represents the average number of individual "infected types" of row i produced by one typical "infected type" of column j in a fully susceptible population of snails and humans (row and column labels are in bold). For example, $E_n \to Cer$ is the average number of cercariae produced by a single snail that is infected as a neonate. Note that we use E instead of I because both snails and humans first enter an exposed class when infected. Variables are as defined in Table1.

Completely Stratified Model NGM Entries

$$Mir \to E_n = e_1 p_1 \frac{S_n}{c}\left[\frac{1}{d_m + e_1\frac{S_n}{c} + e_2\frac{S_j}{c} + e_3\frac{S_s}{c} + e_4\frac{S_l}{c}}\right]$$

$$Mir \to E_j = e_2 p_2 \frac{S_j}{c}\left[\frac{1}{d_m + e_1\frac{S_n}{c} + e_2\frac{S_j}{c} + e_3\frac{S_s}{c} + e_4\frac{S_l}{c}}\right]$$

$$Mir \to E_s = e_3 p_3 \frac{S_s}{c}\left[\frac{1}{d_m + e_1\frac{S_n}{c} + e_2\frac{S_j}{c} + e_3\frac{S_s}{c} + e_4\frac{S_l}{c}}\right]$$

$$Mir \to E_l = e_4 p_4 \frac{S_l}{c}\left[\frac{1}{d_m + e_1\frac{S_n}{c} + e_2\frac{S_j}{c} + e_3\frac{S_s}{c} + e_4\frac{S_l}{c}}\right]$$

$$E_l \to Cer = \left[\frac{y}{y+u_8}\right] s_4 \left[\frac{1}{u_8}\right]$$

$$E_s \to Cer = \left[\frac{y}{y+u_6+\frac{1}{w_3}}\right]\left(s_3\left[\frac{1}{u_6+\frac{1}{w_3}}\right] + \left[\frac{\frac{1}{w_3}}{u_6+\frac{1}{w_3}}\right] s_4 \left[\frac{1}{u_8}\right]\right) + \left[\frac{\frac{1}{w_3}}{y+u_6+\frac{1}{w_3}}\right](E_l \to Cer)$$

$$E_j \to Cer = \left[\frac{y}{y+u_4+\frac{1}{w_2}}\right]\left(s_2\left[\frac{1}{u_4+\frac{l_2}{w_2}}\right] + \left[\frac{\frac{l_2}{w_2}}{u_4+\frac{l_2}{w_2}}\right] s_3\left[\frac{1}{u_6+\frac{1}{w_3}}\right] + \left[\frac{\frac{l_2}{w_2}}{u_4+\frac{l_2}{w_2}}\right]\left[\frac{\frac{1}{w_3}}{u_6+\frac{1}{w_3}}\right] s_4\left[\frac{1}{u_8}\right]\right) + \left[\frac{\frac{1}{w_2}}{y+u_4+\frac{1}{w_2}}\right](E_s \to Cer)$$

$$E_n \to Cer = \left[\frac{y}{y+u_2+\frac{1}{w_1}}\right]\left(s_1\left[\frac{1}{u_2+\frac{l_2}{w_1}}\right] + \left[\frac{\frac{l_2}{w_1}}{u_2+\frac{l_2}{w_1}}\right] s_2\left[\frac{1}{u_4+\frac{l_2}{w_2}}\right] + \left[\frac{\frac{l_2}{w_1}}{u_2+\frac{l_2}{w_1}}\right]\left[\frac{\frac{l_2}{w_2}}{u_4+\frac{l_2}{w_2}}\right] s_3\left[\frac{1}{u_6+\frac{1}{w_3}}\right] + \left[\frac{\frac{l_2}{w_1}}{u_2+\frac{l_2}{w_1}}\right]\left[\frac{\frac{l_2}{w_2}}{u_4+\frac{l_2}{w_2}}\right]\left[\frac{\frac{1}{w_3}}{u_6+\frac{1}{w_3}}\right] s_4\left[\frac{1}{u_8}\right]\right) + \left[\frac{\frac{1}{w_1}}{y+u_2+\frac{1}{w_1}}\right](E_j \to Cer)$$

$$Cer \to E_H = b\frac{S_H}{c}\left[\frac{1}{d_c}\right]$$

$$E_H \to Mir = \left[\frac{l_h}{l_h+u_9}\right]\left(v_1 h_w g_1\left[\frac{1}{q_1+u_9}\right] + v_2 h_w g_2\left[\frac{1}{q_2+u_9}\right] + v_3 h_w g_3\left[\frac{1}{q_3+u_9}\right]\right)$$

3 Sensitivity Analysis Figures

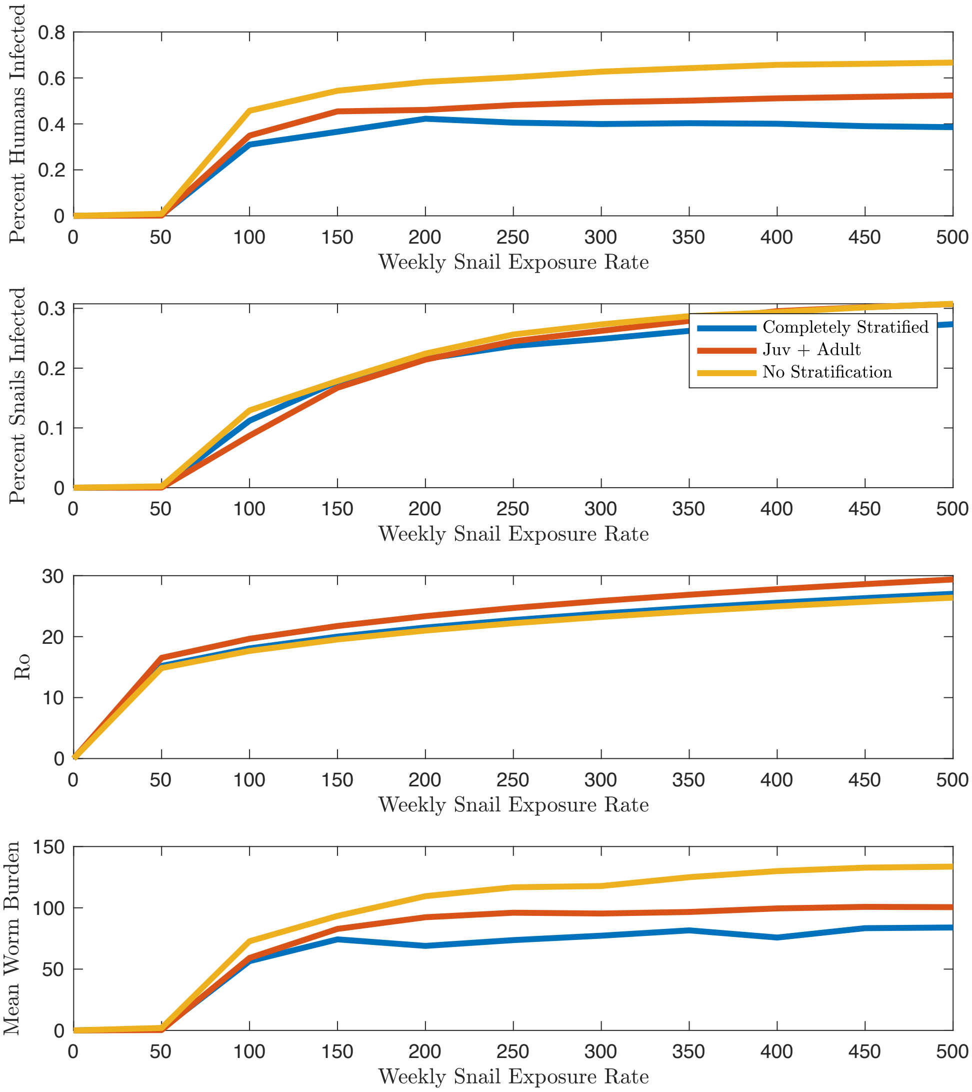

Figure 1: Sensitivity of median human infection prevalence, snail infection prevalence, R_0 and MWB to the exposure rate of snails per week per cercarial density. The median estimates for each model subset were constructed with possible parameter sets from LHS.

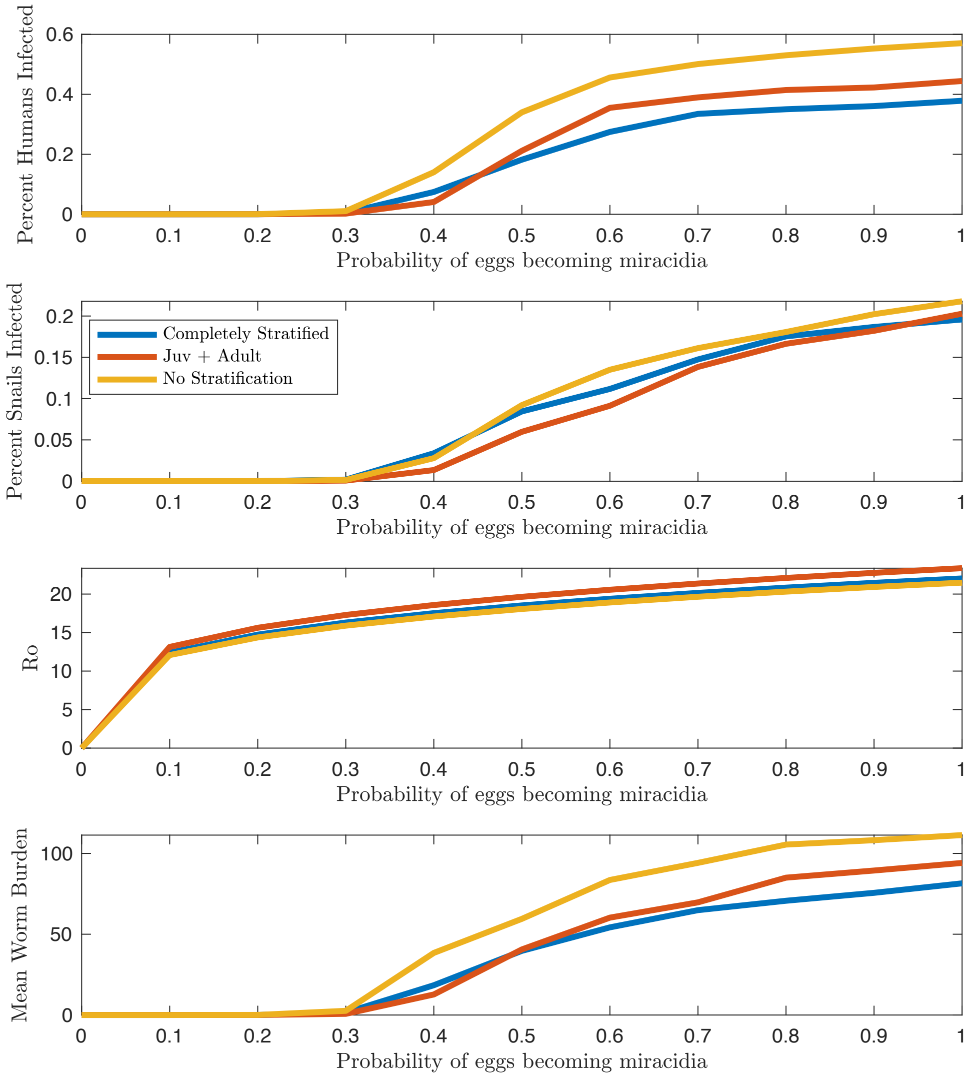

Figure 2: Sensitivity of median human infection prevalence, snail infection prevalence, R_0 and MWB to the probability of eggs becoming miracidia. The median estimates for each model subset were constructed with possible parameter sets from LHS.

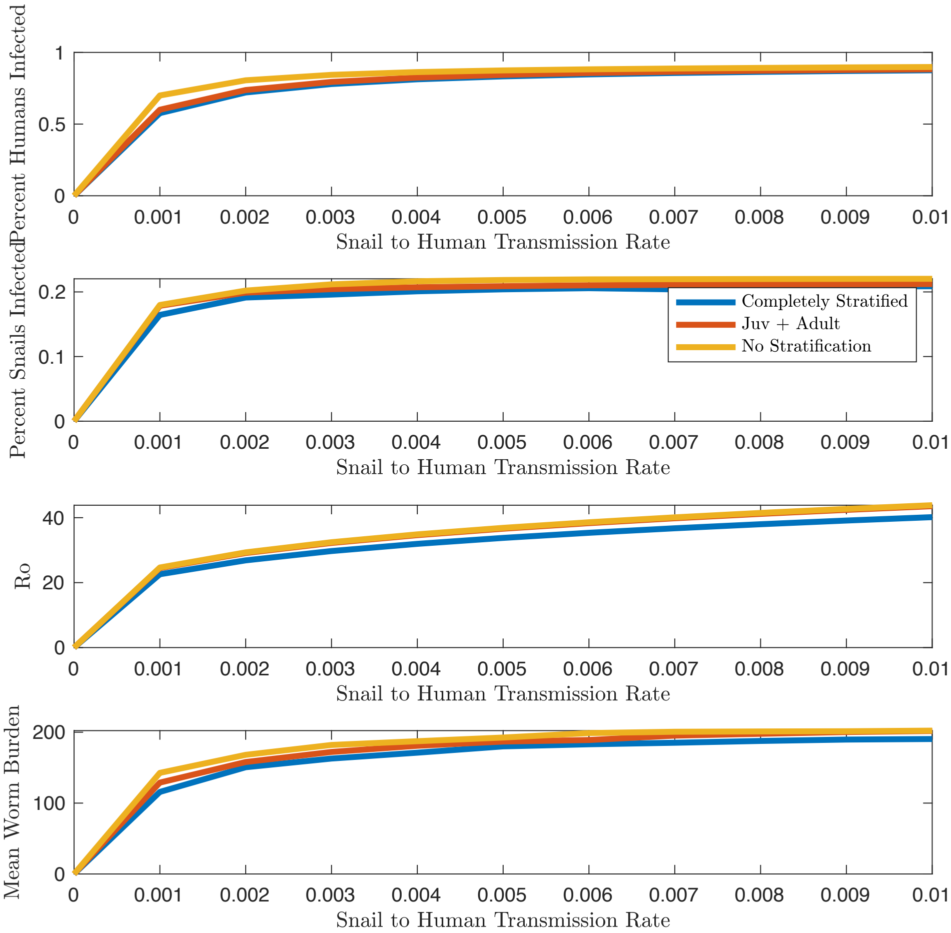

Figure 3: Sensitivity of median human infection prevalence, snail infection prevalence, R_0 and MWB to the snail-to-human transmission rate. The median estimates for each model subset were constructed with possible parameter sets from LHS.

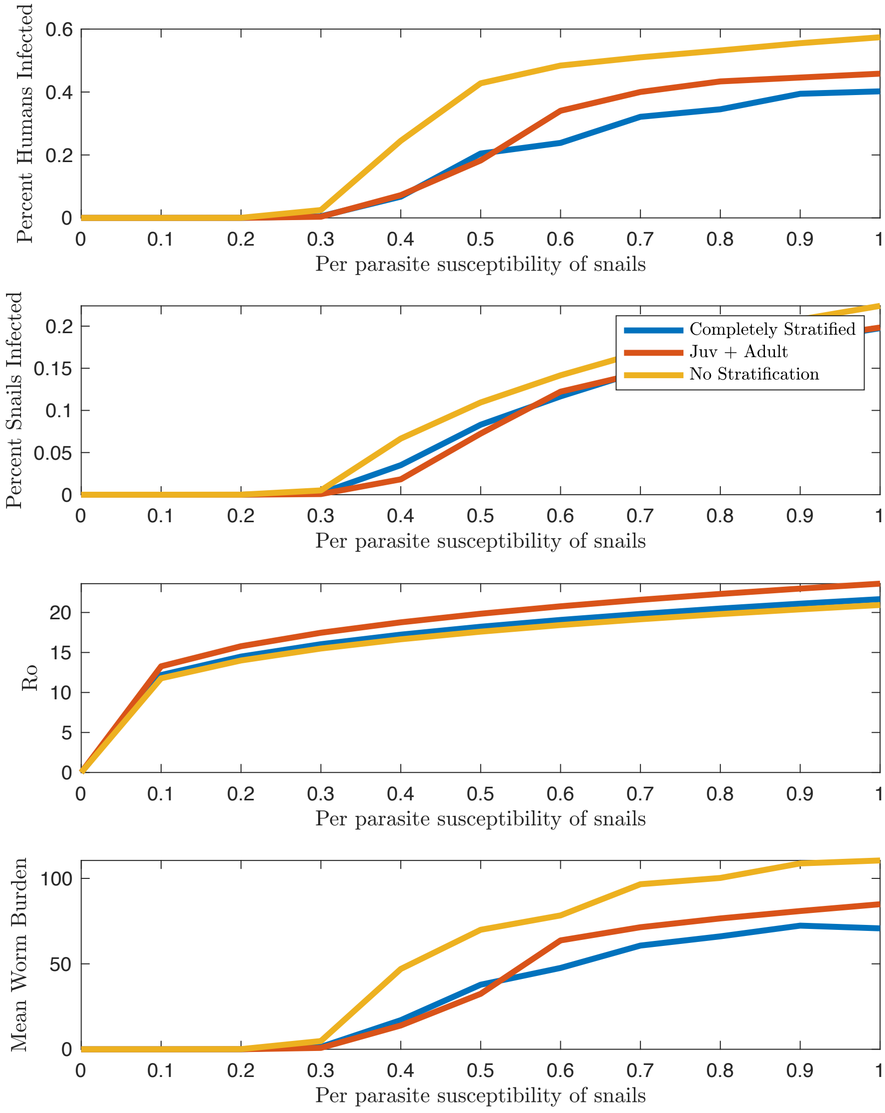

Figure 4: Sensitivity of median human infection prevalence, snail infection prevalence, R_0 and MWB to the per parasite susceptibility of snails. The median estimates for each model subset were constructed with possible parameter sets from LHS.

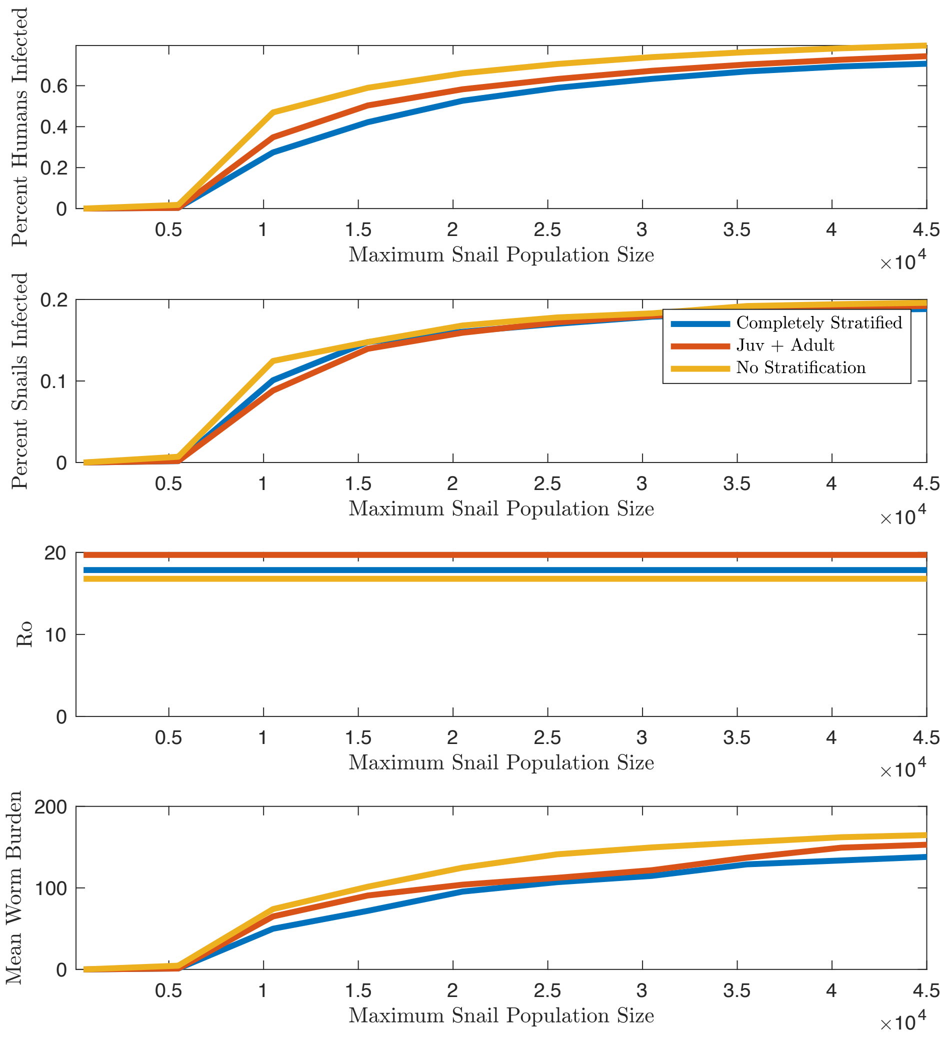

Figure 5: Sensitivity of median human infection prevalence, snail infection prevalence, R_0 and MWB to the maximum snail population size. The median estimates for each model subset were constructed with possible parameter sets from LHS.

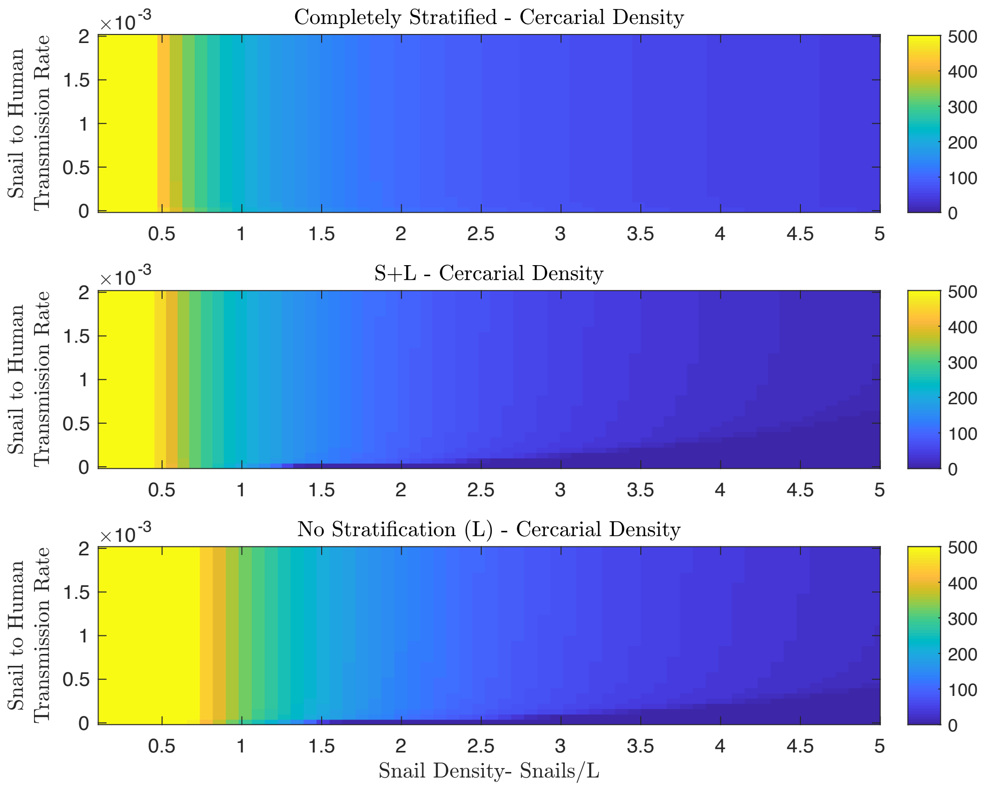

Figure 6: The cercarial density at equilibrium is heavily influenced by the snail density and is less influenced by the snail-to-human transmission rate. Higher cercarial densities are found at similar snail density and snail-to-human transmission rate values for models without age structure than for models with age stratification.

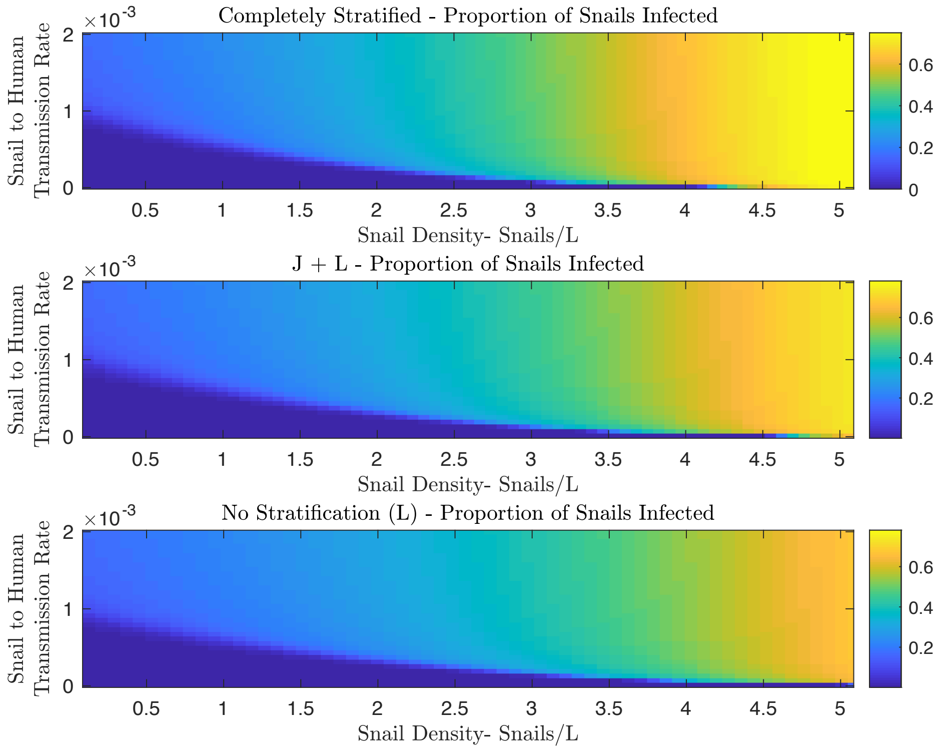

Figure 7: The proportion of infected snails at equilibrium is primarily sensitive to the density of snails with high levels of infection possible only with high snail density, irrespective of the snail-to-human transmission rate. The impact of changes in snail density and snail-to-human transmission rate is consistent over model structure type.

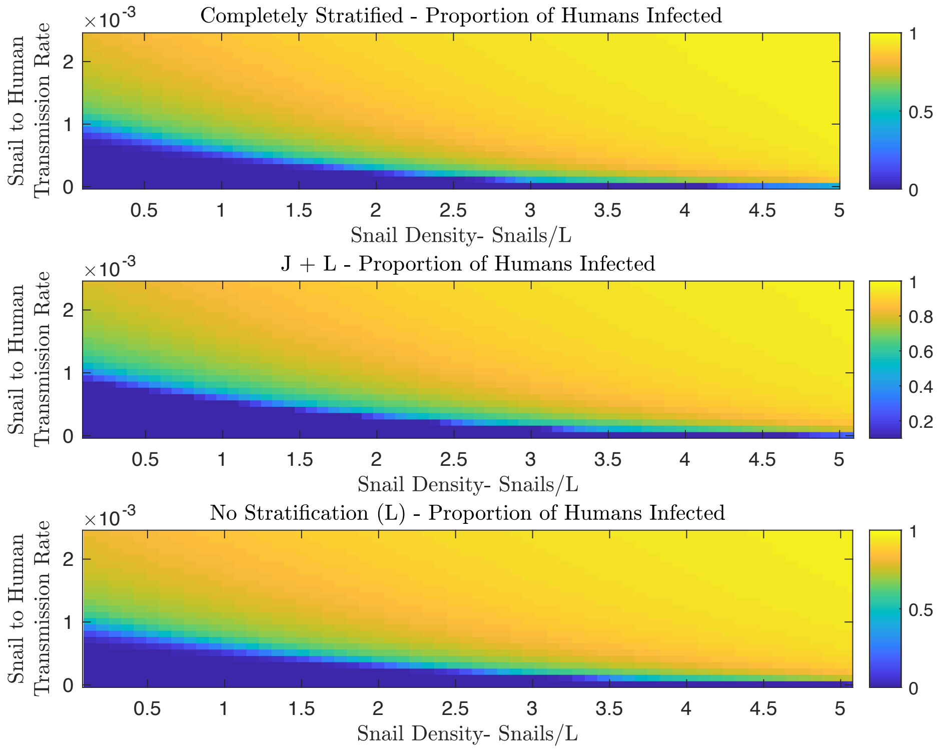

Figure 8: The proportion of humans infected at equilibrium is sensitive to both the snail-to-human transmission rate, and the density of snails. There are greater proportions of humans infected for the model without snail age stratification than for models with snail age stratification at comparable snail density and snail-to-human transmission rate values.

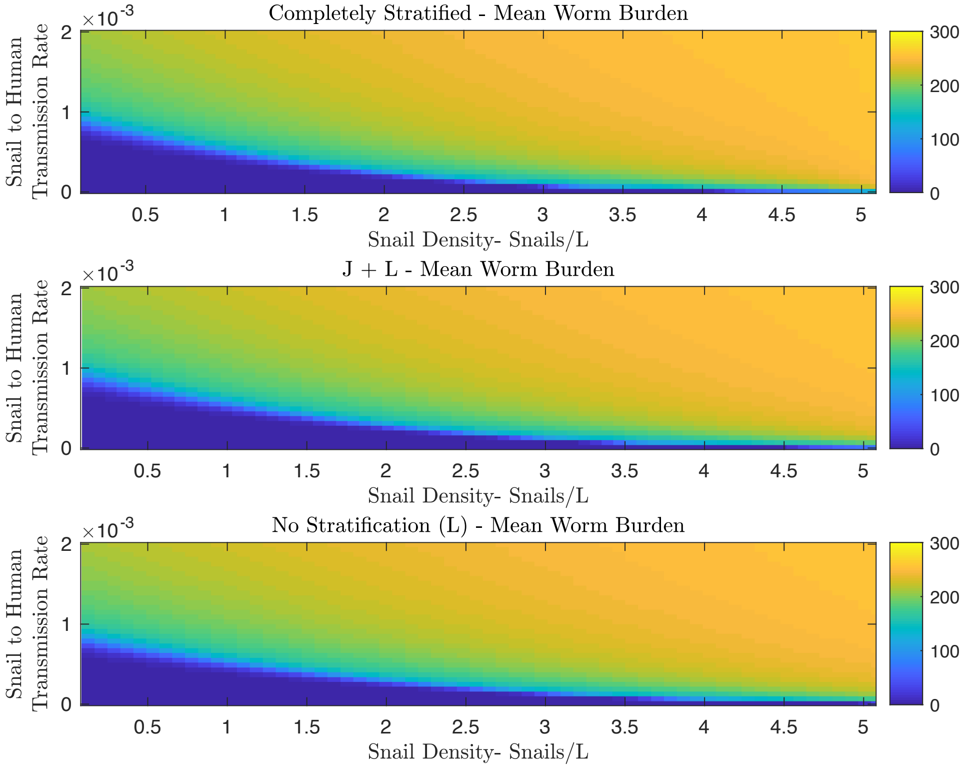

Figure 9: The mean worm burden at equilibrium is sensitive to both the snail-to-human transmission rate, and the density of snails.

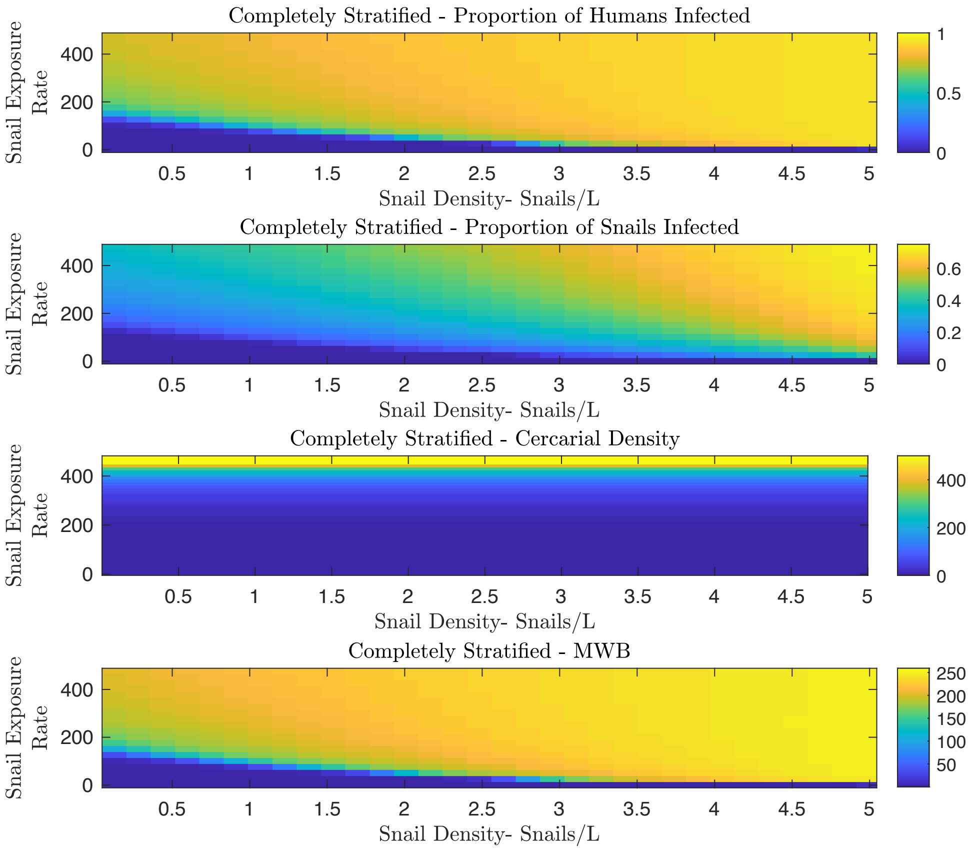

Figure 10: Human infection prevalence, MWB and cercarial density at equilibrium are primarily sensitive to the snail exposure rate over a range of snail densities. The snail infection prevalence is sensitive to both snail exposure rate and snail density

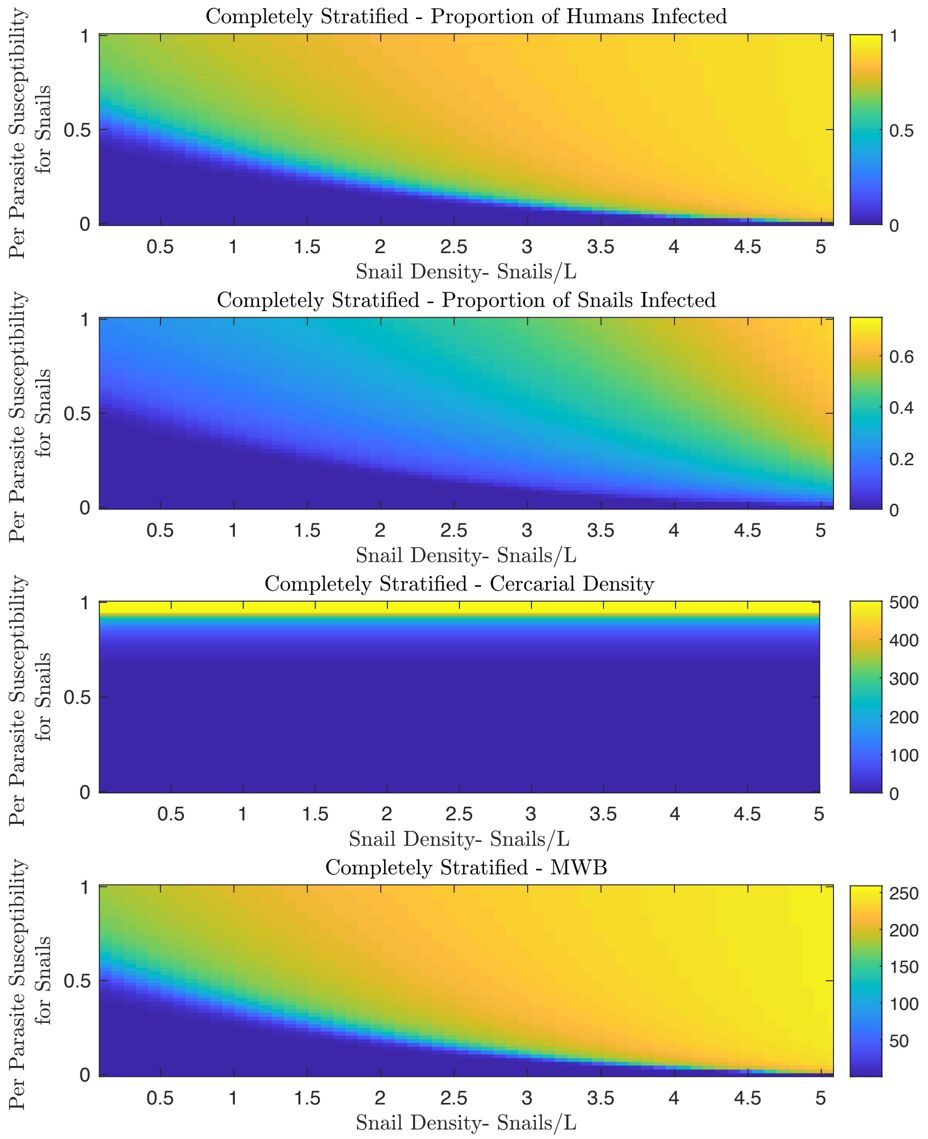

Figure 11: Snail infection prevalence, human infection prevalence and MWB are sensitive to the per parasite susceptibility of snails and snail density. The cercarial density at equilibrium is sensitive primarily to the per parasite susceptibility of snails.

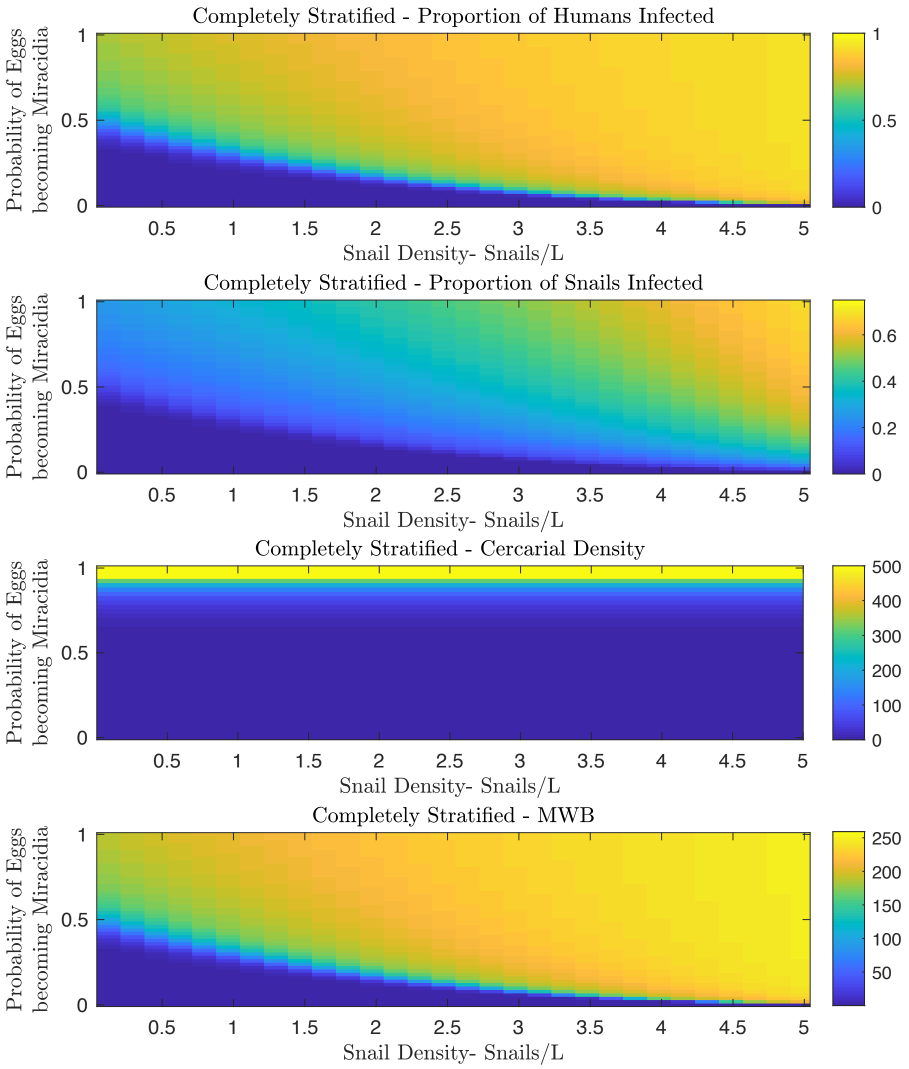

Figure 12: Snail infection prevalence, human infection prevalence and MWB are sensitive to the probability of eggs becoming miracidia and snail density. The cercarial density at equilibrium is sensitive primarily to the probability of eggs becoming miracidia.

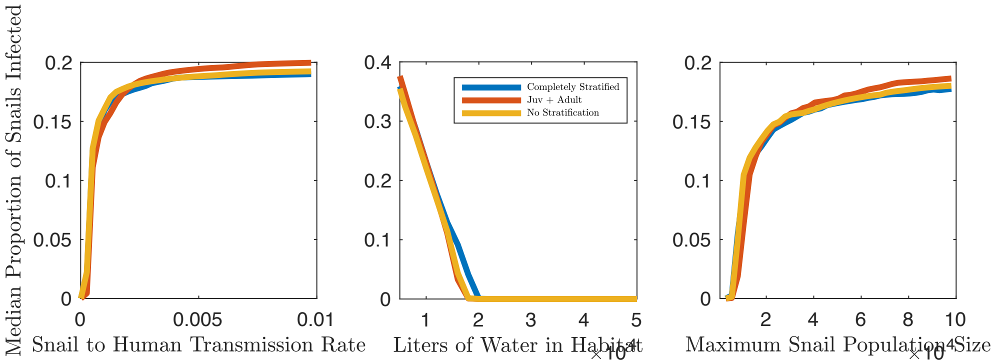

Figure 13: Median snail infection prevalence estimates constructed with LHS over a range of snail-to-human transmission rate values, habitat volume and maximum snail population sizes. All models display comparable median snail infection prevalence estimates over a range of snail-to-human transmission rate values.

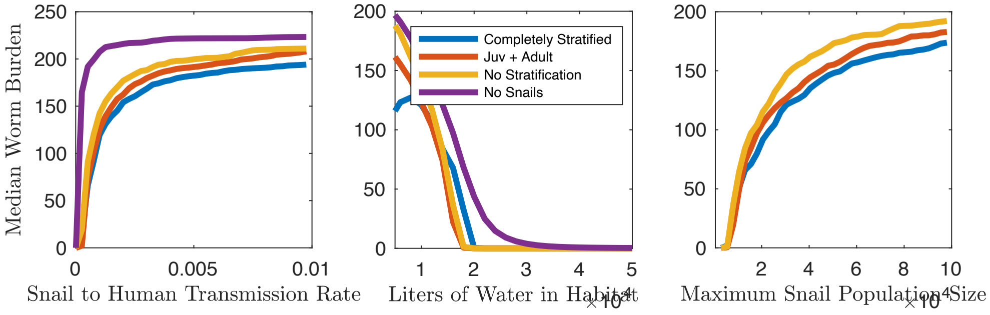

Figure 14: Mean worm burden estimates constructed with LHS over a range of snail-to-human transmission rate values, habitat volume and maximum snail population sizes. Models without snail age stratification display larger mean worm burden estimates over a range of snail-to-human transmission rate values and maximum snail population sizes.

4 R_0 Figures

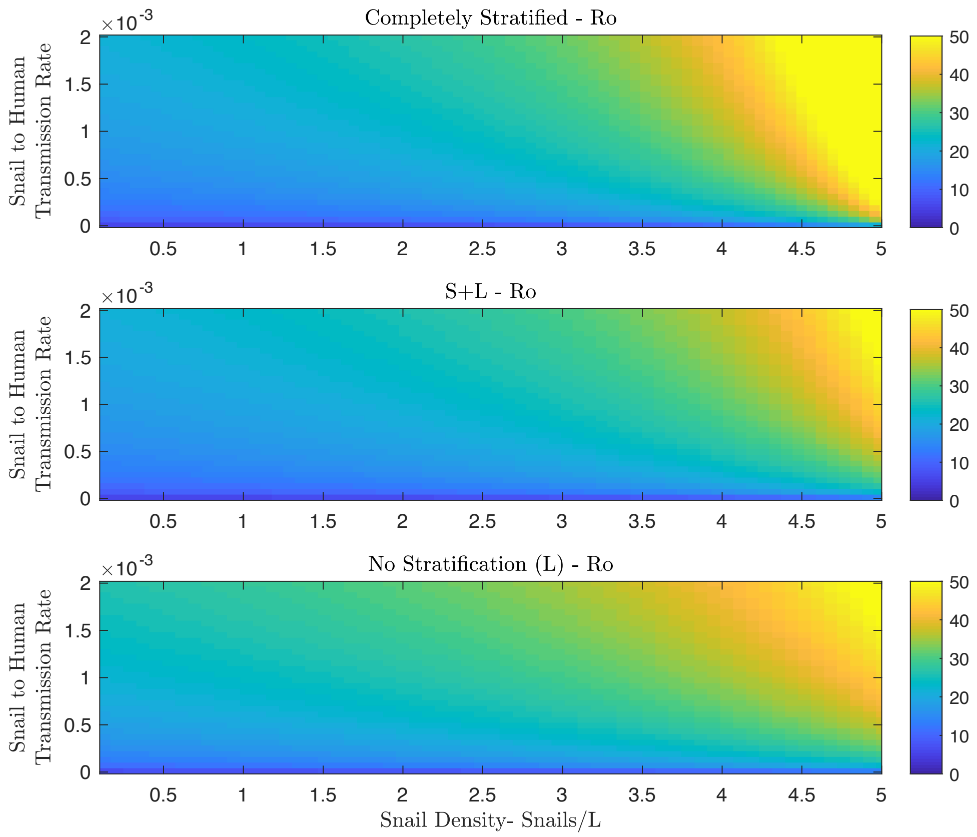

Figure 15: R_0 at equilibrium is sensitive to the snail-to-human transmission rate and snail density and is comparable between models.

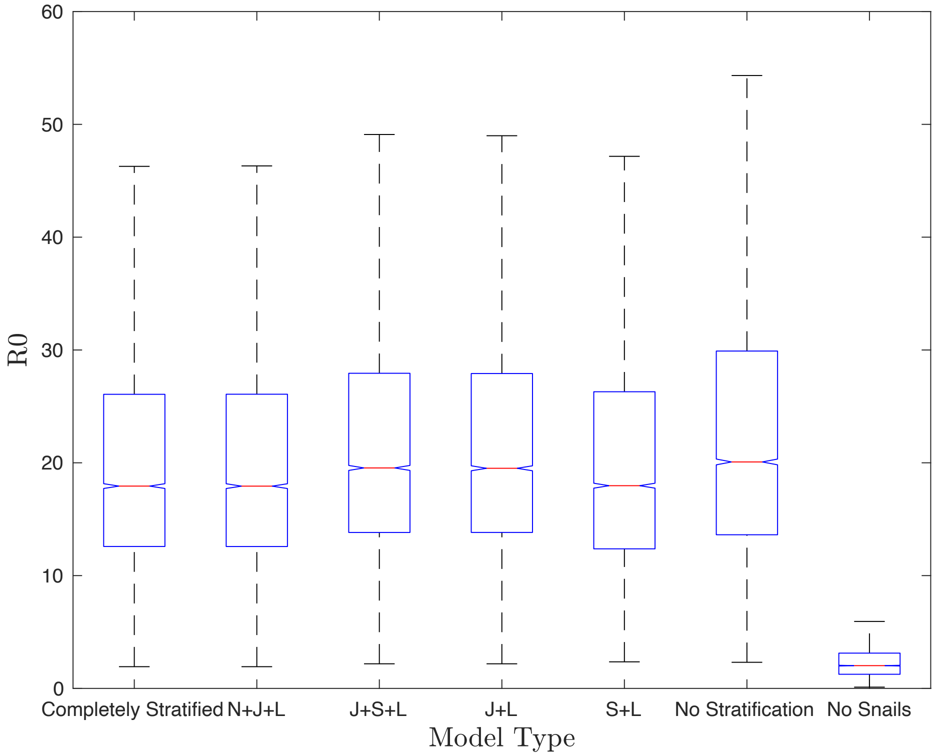

Figure 16: Estimates of R_0 at equilibrium for each model subset with constructed possible parameter sets from Latin Hypercube Sampling (LHS).

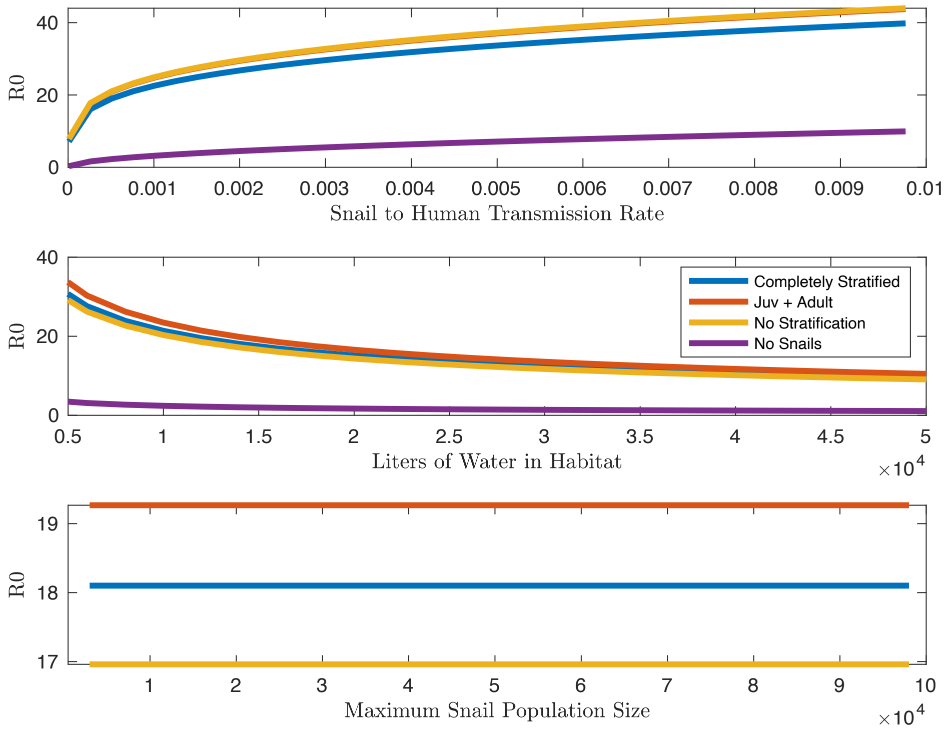

Figure 17: Median R_0 estimates constructed with LHS over a range of snail-to-human transmission rates, habitat volumes and maximum snail population sizes. The models which include snails are comparable over these parameter ranges.

5 Kruskal-Wallis Results

	**Completely Stratified**	**J+S+L**	**N+J+L**	**J+L**	**S+L**	**Unstratified**
**Completely Stratified**	-	-	-	-	-	-
**J+S+L**	2.07E-08	-	-	-	-	-
**N+J+L**	0.9983024	2.07E-08	-	-	-	-
**J+L**	0.9199232	2.70E-08	0.71716	-	-	-
**S+L**	1.05E-07	0.72115	2.67E-08	2.01E-05	-	-
**Unstratified**	0.251051	6.15E-06	0.09874	0.84784	0.00353	-

Table 3: *Snail Infection Prevalence.* The p-values corresponding to Kruskal-Wallis pairwise comparisons for different model options of the median values of snail infection prevalence presented in Figure 6a. Values below .01 are represented in red.

	**Completely Stratified**	**J+S+L**	**N+J+L**	**J+L**	**S+L**	**Unstratified**	**No Snails**
**Completely Stratified**	-	-	-	-	-	-	-
**J+S+L**	3.71E-08	-	-	-	-	-	-
**N+J+L**	2.89E-06	0.0851	-	-	-	-	-
**J+L**	6.92E-08	0.4225	0.9879	-	-	-	-
**S+L**	3.71E-08	3.71E-08	3.71E-08	3.71E-08	-	-	-
**Unstratified**	3.71E-08	3.71E-08	3.71E-08	3.71E-08	0.1420	-	-
**No Snails**	3.71E-08	3.71E-08	3.71E-08	3.71E-08	3.71E-08	3.71E-08	-

Table 4: *Human Infection Prevalence.* The p-values corresponding to Kruskal-Wallis pairwise comparisons for different model options of the median values of human infection prevalence presented in Figure 6b.

	**Completely Stratified**	**J+S+L**	**N+J+L**	**J+L**	**S+L**	**Unstratified**
**Completely Stratified**	-	-	-	-	-	-
**J+S+L**	2.66E-12	-	-	-	-	-
**N+J+L**	4.49E-221	4.49E-221	-	-	-	-
**J+L**	2.28E-91	8.62E-86	4.49E-221	-	-	-
**S+L**	3.93E-138	.2.39E-142	1.88E-102	4.93E-208	-	-
**Unstratified**	4.49E-221	4.49E-221	4.49E-221	4.49E-221	4.08E-70	-

Table 5: *Cercarial Density.* The p-values corresponding to Kruskal-Wallis pairwise comparisons for different model options of the median values cercarial density presented in Figure 6c.

	**Completely Stratified**	**J+S+L**	**N+J+L**	**J+L**	**S+L**	**Unstratified**	**No Snails**
**Completely Stratified**	-	-	-	-	-	-	-
**J+S+L**	4.09E-47	-	-	-	-	-	-
**N+J+L**	4.49E-221	4.49E-221	-	-	-	-	-
**J+L**	2.70E-171	1.45E-177	4.49E-221	-	-	-	-
**S+L**	7.48E-173	3.77E-179	4.28E-159	4.37E-106	-	-	-
**Unstratified**	4.49E-221	4.49E-221	4.49E-221	4.49E-221	1.47E-199	-	-
**No Snails**	1.35E-160	1.24E-173	5.39E-188	2.74E-31	7.62E-41	4.49E-221	-

Table 6: *Mean Worm Burden* The p-values corresponding to Kruskal-Wallis pairwise comparisons for different model options of the median values cercarial density presented in Figure 6c.

	**Completely Stratified**	**J+S+L**	**N+J+L**	**J+L**	**S+L**	**Unstratified**	**No Snails**
**Completely Stratified**	-	-	-	-	-	-	-
**J+S+L**	0.999	-	-	-	-	-	-
**N+J+L**	3.71E-08	3.71E-08	-	-	-	-	-
**J+L**	3.706E-08	3.706E-08	3.706E-08	-	-	-	-
**S+L**	3.706E-08	0.9999	3.706E-08	3.706E-08	-	-	-
**Unstratified**	3.706E-08	3.706E-08	0.0743	0.0673	3.706E-08	-	-
**No Snails**	3.706E-08	3.706E-08	3.706E-08	3.706E-08	3.706E-08	3.706E-08	-

Table 7: R_0. The p-values corresponding to Kruskal-Wallis pairwise comparisons for different model options of the median values of R_0 presented in Supplementary Figure 10.

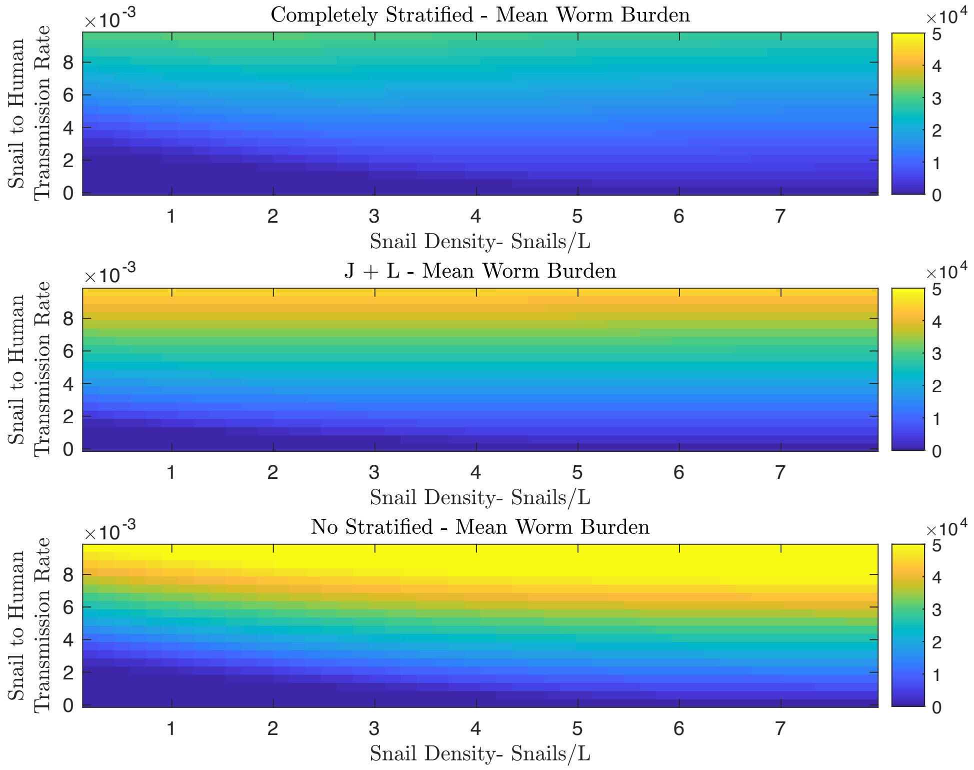

Figure 18: Mean worm burden as calculated in the method of the model of Anderson and May. In this model MWB is sensitive to the snail-to-human transmission rate with high levels of infection possible only with high snail density, irrespective of the density of snails. The models with age stratification display lower MWB than the unstratified model.

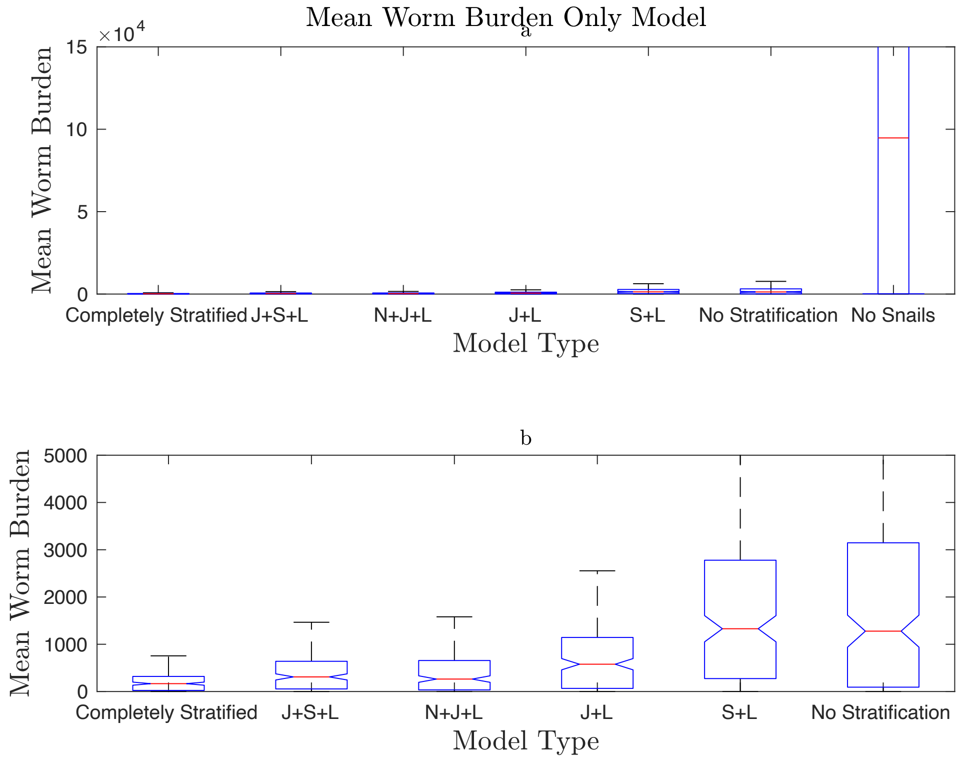

Figure 19: Mean worm burden as calculated in the method of the model of Anderson and May at equilibrium for each model subset with constructed possible parameter sets from LHS. Panel a: The mean worm burden in models lacking snails was substantially higher than for those models containing snails. Panel b: The estimates varied over the different model stratification configurations containing snails.
